# Exploring the Antimicrobial and Antiviral Properties of Cryptic Peptides from Human Fibrinogen

**DOI:** 10.3390/ijms26188914

**Published:** 2025-09-12

**Authors:** Andrea Bosso, Antonio Masino, Ilaria Di Nardo, Carla Zannella, Rosa Gaglione, Ida Palumbo, Rosanna Culurciello, Anna De Filippis, Marcelo D. T. Torres, Cesar de la Fuente-Nunez, Massimiliano Galdiero, Angela Arciello, Antimo Di Maro, Elio Pizzo, Valeria Cafaro, Eugenio Notomista

**Affiliations:** 1 Department of Biology, University of Naples Federico II, Via Vicinale Cupa Cintia, 26, 80126 Naples, Italy; antonio.masino@unina.it (A.M.); ilaria.dinardo@unina.it (I.D.N.); ida.palumbo2@unina.it (I.P.); rosanna.culurciello@unina.it (R.C.); elipizzo@unina.it (E.P.); vcafaro@unina.it (V.C.); notomist@unina.it (E.N.); 2 Department of Translational Medical Science, University of Naples Federico II, 80131 Naples, Italy; 3 ImmunoNutritionLab at CEINGE Advanced Biotechnologies, University of Naples Federico II, 80131 Naples, Italy; 4 Department of Experimental Medicine, University of Campania “Luigi Vanvitelli”, 80138 Napoli, Italy; carla.zannella@unicampania.it (C.Z.); anna.defilippis@unicampania.it (A.D.F.); massimiliano.galdiero@unicampania.it (M.G.); 5 Department of Chemical Sciences, University of Naples Federico II, 80126 Naples, Italy; rosa.gaglione@unina.it (R.G.); anarciel@unina.it (A.A.); 6 Machine Biology Group, Departments of Psychiatry and Microbiology, Institute for Biomedical Informatics, Institute for Translational Medicine and Therapeutics, Perelman School of Medicine, University of Pennsylvania, Philadelphia, PA 19104, USA; marcelot@upenn.edu (M.D.T.T.); cfuente@upenn.edu (C.d.l.F.-N.); 7 Departments of Bioengineering and Chemical and Biomolecular Engineering, School of Engineering and Applied Science, University of Pennsylvania, Philadelphia, PA 19104, USA; 8 Department of Chemistry, School of Arts and Sciences, University of Pennsylvania, Philadelphia, PA 19104, USA; 9 Penn Institute for Computational Science, University of Pennsylvania, Philadelphia, PA 19104, USA; 10 Department of Environmental, Biological and Pharmaceutical Sciences and Technologies (DiSTABiF), University of Campania ‘Luigi Vanvitelli’, Via Vivaldi 43, 81100 Caserta, Italy; antimo.dimaro@unicampania.it; 11 Centro Servizi Metrologici e Tecnologici Avanzati (CeSMA), Complesso Universitario di Monte Sant’Angelo, Via Cinthia 21, 80126 Naples, Italy

**Keywords:** antimicrobial peptides, host defense peptides, cryptic peptides, fibrinogen, thrombin, anti-biofilm, LPS-inhibition, anti-viral activity

## Abstract

Fibrinogen (FIB), a key component of the coagulation cascade, is traditionally recognized for its role in hemostasis and tissue repair. However, due to its high plasma abundance and susceptibility to proteolytic cleavage during inflammation, it may also represent a previously unrecognized source of bioactive peptides. This study presents, for the first time, a comprehensive analysis of the antimicrobial, anti-inflammatory, and antiviral properties of six cationic antimicrobial peptides (AMPs) deriving from the C-terminal extremities of the three subunits of human fibrinogen (FIBα, FIBβ, and FIBγ), identified using a scoring function developed by our group. Antibacterial assays against Gram-positive and Gram-negative pathogens revealed different antimicrobial activity profile depending on their parent protein. Selected peptides displayed additive or synergistic effects when combined with conventional antibiotics or the thrombin-derived peptide (P)GKY20, highlighting their potential for combination therapies. Hemolytic assay confirmed the biocompatibility of fibrinogen-derived cryptic peptides with erythrocytes. Furthermore, the peptides significantly reduced LPS-induced nitric oxide release in murine macrophages Raw 264.7 cells, indicating anti-inflammatory activity. Notably, antiviral activity was observed against enveloped viruses (HCoV-229E and HSV-1) under various treatment conditions, while no activity was detected against the non-enveloped virus CVB3. Overall, these findings reveal human fibrinogen as a source of multifunctional cryptic peptides with broad-spectrum antimicrobial, antiviral, and immunomodulatory activities, supporting their potential as part of the innate immune system.

## 1. Introduction

Antimicrobial peptides (AMPs) constitute fundamental components of the innate immune system and are present in various organisms ranging from bacteria, plants, invertebrates and vertebrates. In vertebrates, they are not only endowed with direct antimicrobial and antibiofilm activities but also display endotoxin-scavenging and immune-modulating functions, that allow these peptides to act as mediators between innate and adaptive immunity [1,2,3,4]. Defensins and cathelicidins, the most well-known vertebrate AMPs, are coded by dedicated genes directing the synthesis of precursors that undergo proteolytic maturation during the secretion process [5,6,7]. Over the past decade, increasing interest has emerged around an underexplored class of antimicrobial peptides that have been called “cryptic”, “encrypted”, or also “intragenic” because they are hidden withinthe sequence of larger precursor proteins not necessarily related to host defense [8,9,10,11]. Upon proteolytic cleavage of host proteins, these cryptic peptides are released and display all the typical biological activities of natural AMPs [9,12,13]. A surprisingly wide panel of proteins with different functions hosts such peptides: proteases of the coagulation cascade (including thrombin), chemokines and cytokines, RNases and lysozymes, lactoferrin, hemoglobin, albumin, pepsinogen and apolipoproteins, just to mention some of the most well-characterized [8,14,15,16,17,18,19,20].

The great interest in cryptic peptides and their precursor proteins is motivated by the fact that (i) precursor proteins represent a previously unexploited reservoir of potential antimicrobials to combat pathogenic bacteria (e.g., multidrug-resistant strains) [21,22,23] and (ii) a thorough inventory of these peptides would allow to better understand a potentially overlooked aspect of the immune system. In this regard, the direct in vivo antimicrobial efficacy of natural AMPs has been questioned because their concentration is often lower than the MIC (minimal inhibitory concentration) values [24,25]. However, the antimicrobial activity of AMPs and cryptic peptides is generally additive or synergistic [26,27,28], therefore, demonstrating the existence of many cryptic peptides capable of cooperating with conventional AMPs would help to resolve this apparent paradox.

Identifying novel cryptic peptides using traditional techniques (e.g., controlled proteolysis followed by isolation and characterization of the fragments) is costly in terms of time and resources [29,30]. In silico identification can speed up identification, however, most of the available tools for the identification of AMPs [31,32] are not specifically designed to search for such peptides. Our group has developed a sliding window method that allows to detect the position of cryptic AMP-like regions inside protein sequences by calculating, for each peptide, a score that depends on the similarity of the aminoacidic composition of the peptide to the aminoacidic composition typical of membrane-targeting AMPs [33,34]. These are the most common type of AMPs (e.g., cathelicidins generally belong to this class) and the most interesting one, because by targeting the lipidic structure of membranes rather than a specific target like a protein or ribosomes, they rarely give rise to resistant strains [35,36,37]. Our method, trustworthy while computationally not demanding and inexpensive, allowed to identify tens of new cryptic peptides in human, plant and even prokaryotic proteins [8,38,39,40]. More recently, in Torres et al. 2022 [41], the method has been improved and used to screen the whole human proteome allowing to identify several hundreds of hypothetical new cryptic AMPs. Several tens of these peptides were synthesized, and their antimicrobial activity was demonstrated in vitro. The efficacy of two peptides derived from platelet proteins was also demonstrated in vivo, in a murine model of skin infection.

Here, we report for the first time the in-depth biological characterization of six cryptic peptides derived from the C-termini of the subunits α, β and γ of human fibrinogen (FIBα, FIBβ, and FIBγ). The interest for this protein is justified by several reasons: (i) fibrinogen is one of the most abundant proteins in human plasma after albumin, reaching concentrations as high as 3 mg/mL [42,43]; (ii) it plays a pivotal role in the coagulation being rapidly converted to fibrin by thrombin during vascular injury, thereby initiating clot formation and tissue repair [44]; (iii) conversion to fibrin does not involve the C-termini, thus fibrin still hosts the antimicrobial regions; (iv) fibrinogen, fibrin and their degradation products are implicated in several physiological and pathological processes, including inflammation, wound healing, and host defense [45,46]; (v) being exposed to proteolytic processing during inflammation and coagulation [47,48], fibrinogen and fibrin represent plausible candidates as a source of bioactive fragments which would coexist and cooperate with the well-known cryptic peptides released by thrombin and the other proteases of the coagulation cascade [46,49]. The characterization of the six human fibrinogen-derived peptides, produced both in the recombinant form and by chemical synthesis, confirmed that, to a different degree, they all show promising biological activities comparable to those of vertebrate cathelicidins and cathelicidin-like cryptic peptides.

## 2. Results and Discussion

### 2.1. In Silico Analysis of Human Fibrinogen

**Figure 1:**
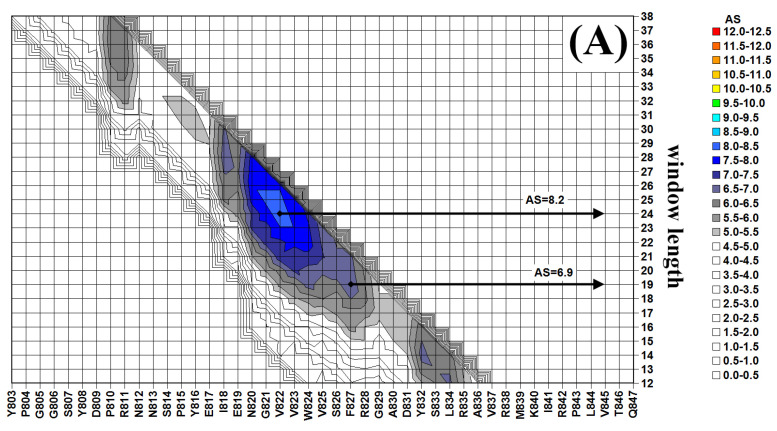

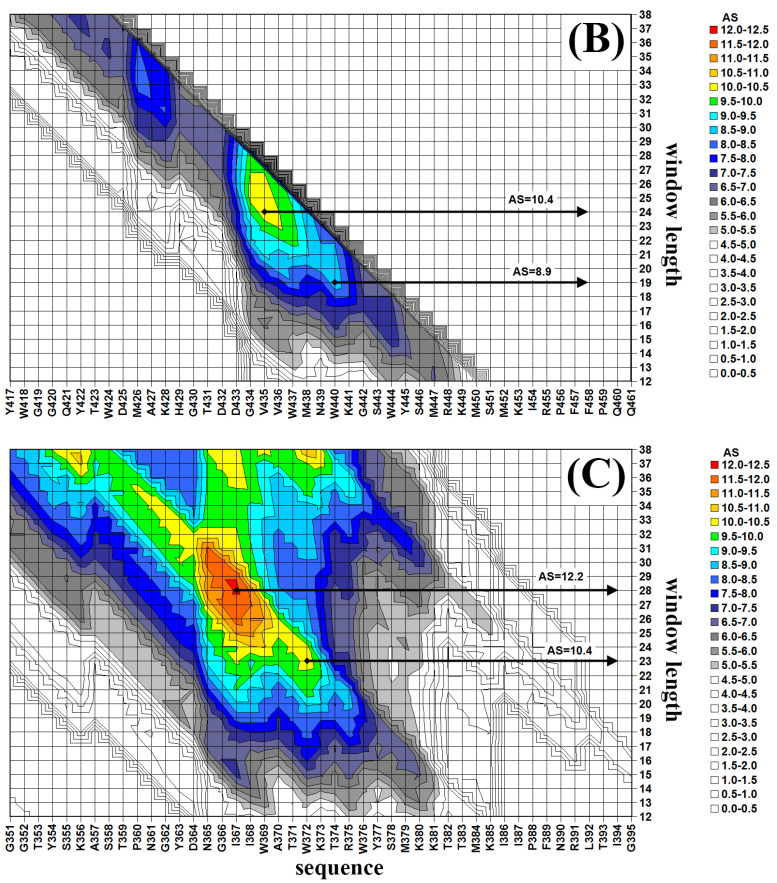
Sliding window analysis of the C-terminal region of the FIB subunits. (**A**) FIBα (**B**) FIBβ (**C**) FIBγ. Sequence and window length are reported on the x and y axis, respectively. The arrows indicate the position of the absolute maxima and of the relative maximum located inside the region of the absolute maximum.

**Figure 2:**
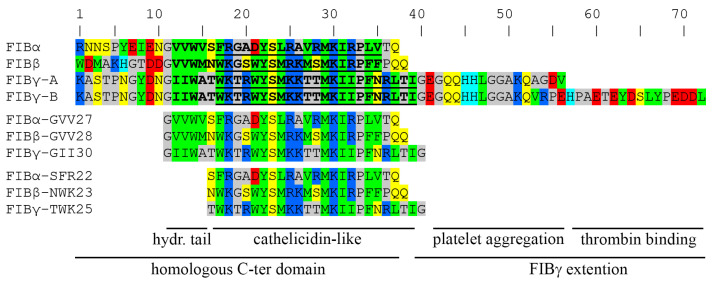
Sequence alignment of the FIB subunits C-termini and of the FIB-derived cryptic peptides. The sequences corresponding to the AS and relative maxima are shown in bold and bold/underlined, respectively. Residues are colored according to their chemical properties: red—acidic; blue—basic, green—hydrophobic; yellow—hydrophilic uncharged; gray—borderline; cyan—histidine.

Unlike most in silico tools for the identification of AMPs and cryptic peptides, the tool developed by our group can be used not only to identify putative cryptic antimicrobial regions but also to perform a detailed local analysis of the sequence and to identify the shortest active fragments. Figure 1 shows the sliding window analysis of the C-terminal regions of human fibrinogen subunits. The contour plots report the Absolute Score (AS) values of each peptide as a function of its starting residue (x axis) and peptide length (y axis). AS values are correlated to antimicrobial activity: values higher than 6 correspond to measurable activity; antimicrobial activity increases linearly for AS values between 6 and 10–11; for higher values linearity is lost, i.e., further increases in the AS values are not necessarily associated with a significant reduction in MIC values. The contour plots of fibrinogen subunits, clearly show the presence of an absolute maximum starting at residues FIBα V822 (24 aa), FIBβ V435 (24 aa) and FIBγ I367 (28 aa). The alignment shown in Figure 2 highlights the similarity among the three peptides. This is not surprising as the C-terminal domains of the fibrinogen subunits are homologous, however, a few differences in the number of positively charged and hydrophobic residues determine significant differences in the AS values of the three cryptic peptides. It should also be noted that, in the case of subunits α and β, the high scoring regions are exactly at the C-terminus (Figure 2), whereas, in the case of the FIBγ, due to the presence of an extension not present in the other two subunits, the high scoring region is 17 or 33 residues upstream the C-terminus (Figure 2) depending on the alternative splicing isoform of FIBγ, FIBγ-A and FIBγ-B (also known as FIBγ’), respectively. Very interestingly these extensions have been implicated in defense and clotting [50,51]. In particular, the C-terminal sequence HHLGGAKQAGDV of FIBγ-A is involved in platelet aggregation [52,53], whereas the acidic region at the C-terminus of FIBγ-B mediates the high affinity binding of fibrinogen to thrombin [54]. The contour plots also show the presence of a lower relative maximum at the end of a narrow crest on the left of the main peak and corresponding to residues FIBα F827 (19 aa), FIBβ W440 (19 aa) and FIBγ W372 (23 aa). It is interesting to note that the peptides corresponding to the absolute and local maxima differ for the presence of an N-terminal stretch of 4 consecutive hydrophobic/β-preferring residues: VVWV (FIBα), VVWM (FIBβ) and IIWA (FIBγ) (Figure 2). As it is well known that such sequences are often associated with the propensity to aggregate or to form amyloid structures, the C-termini of FIB subunits were also analyzed by using three alternative tools for the identifications of aggregation prone regions in proteins, namely TANGO [55,56], AmylPRED [57] and AggreSCAN [58], which confirmed the presence of aggregation-prone regions (6–8 residues long) including to the above-mentioned hydrophobic stretches (see Supplementary file S1: Aggregation Propensity Analysis). As propensity to aggregate or to form amyloid fibers could influence the biological properties of the peptides, both the longer and the shorter form of each peptide were selected for a comprehensive experimental characterization. The sequences of the six peptides here analyzed, FIBα-GVV27, FIBα-SFR22, FIBβ-GVV28, FIBβ-NWK23, FIBγ-GII30 and FIBγ-TWK25, are shown in Figure 2. It should be noted that the selected peptides include few hydrophilic residues flanking the absolute and relative maxima identified through the contour plots: T486-Q487 and P459-Q460-Q461 are the last residues of FIBα and FIBβ, respectively, so they were included in the peptides FIBα-GVV27, FIBα-SFR22, FIBβ-GVV28 and FIBβ-NWK28; the single Gly, Ser, Thr, and Asn residues at the N-termini of all the FIB derived-peptides and at the C-termini of FIBγ derived peptides were included based on the hypothesis that small, uncharged residues could enhance the interaction between the positively charged termini and the phospholipid heads, thereby improving membrane binding.

### 2.2. Recombinant Expression FIB-Derived Peptides

The six peptides were produced in the recombinant form by using a previously described strategy that exploit the frog protein onconase (ONC) as a carrier to drive the peptide to inclusion bodies [39,59]. The fusion proteins were expressed in *Escherichia coli* strain BL21DE3, using the pET22b(+) vector and named: ONC-DCless-H6-(P)FIBα-GVV27, ONC-DCless-H6-(P)FIBα-SFR22, ONC-DCless-H6-(P)FIBβ-GVV28, ONC-DCless-H6-(P)FIBβ-NWK23, ONC-DCless-H6-(P)FIBγ-GII30, ONC-DCless-H6-(P)FIBγ-TWK25 (nucleotide and amino acid sequences are reported in Supplementary Figure S1). Fusion proteins were effectively expressed in *E. coli* (140–160 mg/L of LB culture) as inclusion bodies (Supplementary Figure S2) and purified by immobilized metal ion affinity chromatography (IMAC) in denaturing conditions (see Methods section for details). Peptides were released by hydrolysis of an acidic labile “Asp-Pro” sequence leading to recombinant peptides endowed with an additional Pro residue at the N-terminus. Thus, the recombinant forms of these peptides are herein named (P)FIBα-GVV27, (P)FIBα-SFR22, (P)FIBβ-GVV28, (P)FIBβ-NWK23, (P)FIBγ-GII30, (P)FIBγ-TWK25. Hydrolysis efficiencies of 95% were estimated for all the recombinant proteins (Supplementary Figure S3). To purify the recombinant peptides from the hydrolysis mixtures, the carrier and the uncleaved fusion protein were selectively precipitated by increasing the pH to 7 exploiting the fact that ONC is insoluble at pH higher than 5, whereas most AMPs are soluble also at neutral pH. Unfortunately, two peptides, (P)FIBβ-GVV28 and (P)FIBγ-GII30, proved to be particularly prone to aggregation and precipitation in agreement with the in-silico analysis, with low recovery in the soluble phase particularly in the case of (P)FIBγ-GII30. Therefore, purification of (P)FIBα-SFR22, (P)FIBα-GVV27, (P)FIBβ-NWK23 and (P)FIBγ-TWK25 was first performed by selective precipitation, to remove the carrier and the uncleaved fusion protein, followed by Reverse Phase High-Performance Liquid Chromatography (RP-HPLC) on a C18 column, whereas purification of (P)FIBβ-GVV28 and (P)FIBγ-GII30 was performed directly by RP-HPLC omitting the selective precipitation step. Final yields of all recombinant peptides ranged from approximately 6 to 12 mg per liter of LB culture. Peptide purity, as determined by RP-HPLC, typically varied between 95% and 99% (Supplementary Figure S4). These results confirm that recombinant expression in *E. coli* represents a highly efficient strategy for producing recombinant fibrinogen derived cryptic AMPs suitable for a possible scale up [60].

### 2.3. Antimicrobial Activity

The antimicrobial activity of the recombinant FIB-derived peptides was evaluated and compared to that of synthetic counterparts without the initial proline on a panel of Gram-negative and Gram-positive strains including strains belonging to the so-called ESKAPEE pathogens [61,62,63]. (P)GKY20, the most active among the human thrombin-derived cryptic antimicrobial peptides [59], vancomycin and polymyxin B were used as controls. Synthetic and recombinant FIB-derived peptides showed essentially identical antimicrobial activity, in fact, minimal inhibitory concentrations (MIC) values were very similar with differences not greater than a single scalar dilution (Table 1), thus confirming that the additional proline in recombinant peptides has no effect on the antimicrobial activity.

In general, the observed MIC values are in good agreement with the in silico prediction, in fact, according to the AS values, the expected MIC values should be in the order:

FIBγ-GII30 > FIBγ-TWK25 ≅ FIBβ-GVV28 > FIBβ-NWK23 > FIBα-GVV27 > FIBα-SFR22,

whereas the experimental values on the ten analyzed strains are on average in the order:

FIBγ-TWK25 > FIBγ-GII30 ≅ FIBβ-GVV28 ≅ FIBβ-NWK23 > FIBα-GVV27 > FIBα-SFR22.

The fact that the MIC values of FIBγ-GII30 are lower than those of FIBγ-TWK25 and those of FIBβ-GVV28 are very similar to those of FIBβ-NWK23 might be due to the propensity to aggregate of the two longer peptides that could in turn decrease their bioavailability and, hence, their antimicrobial activity. Moreover, it is worth noting that the MIC values of (P)FIBγ-TWK25 are comparable to those of the reference peptide (P)GKY20, thus suggesting that (P)FIBγ-TWK25 could be a promising candidate for biomedical antimicrobial applications.

Next, we measured the Minimum Bactericidal Concentration (MBC) of FIB-derived peptides to determine if they are bactericidal or bacteriostatic agents. Typically, antibacterial agents are classified as bactericidal when the MBC does not exceed four times the MIC. As controls, we tested (P)GKY20, human cathelicidin LL-37, colistin, vancomycin, tobramycin and ciprofloxacin. As reported in Table 2, all tested AMPs showed MBC/MIC ratios of 1 on both *P. aeruginosa* PAO1 and *S. aureus*, thus indicating that FIB-derived peptides exhibit a clear bactericidal activity, similarly to (P)GKY20 and LL-37.

### 2.4. Synergistic Properties

Fibrinogen is not only a key factor in coagulation but also accumulates in the form of fibrin at wound sites, where microbial invasion is likely [45,64]. At these sites several FIB-derived peptides could coexist with cryptic peptides derived from other coagulation factors like thrombin. Their co-localization and potential synergistic effects could enhance the overall antimicrobial response, reducing the concentration of each peptide required for activity. From a therapeutic point of view, a very interesting strategy would be to use synergistic combinations of AMPs and conventional antibiotics [65,66,67,68] that could increase efficiency, decrease the likelihood of resistance onset and minimize cytotoxicity.

Therefore, we tested potential synergistic interactions between couples of FIB-derived peptides as well as between combinations of FIB-derived peptides, thrombin-derived peptide (P)GKY20 and conventional antibiotics (i.e., colistin, tobramycin, and ciprofloxacin). To this purpose, Fractional Inhibitory Concentration Indexes (FICI) on *P. aeruginosa* PAO1 and *S. aureus* were determined by using checkerboard assay (Table 3) [69].

According to three largely used interpretation models [70,71,72], FICI values ≤ 0.5 indicate full synergy, whereas FICI > 1 indicate that there is no significant interaction between the two antimicrobials. On the contrary, in the case of FICI values between 0.5 and 1, there is no agreement among the models: only Fratini’s model [71] considers the values that fall within this range as an indication of synergy, whereas, according to the most stringent models [70,72], these values should be considered more appropriately as an indication of additivity.

When peptide combinations were tested, we found FICI ≤ 1 thus indicating at least an additive behavior. However, the couple (P)FIBγ-GII30 + (P)GKY20 showed FICI ≤ 0.5, both in the case of *P. aeruginosa* and *S. aureus*, thus indicating a strong synergistic behavior for these two peptides. Moreover, several peptide combinations showed FICI values higher but very close to the 0.5 threshold (numbers in bold on green background in Table 3).

In the case of the peptide/antibiotic combinations the picture is more complex. Very interestingly all the peptides/colistin combinations showed FICI values well below 0.5 (in the range 0.18–0.31) thus indicating full synergy. In the case of tobramycin, we always found FICI ≤ 1 thus indicating at least additivity, and FICI values in the range 0.5–0.53 for three peptides (namely FIBγ-GII30 and the FIBβ-derived peptides) on *P. aeruginosa*, indicating synergy. Finally, in the case of ciprofloxacin, we found FICI = 0.75–1 (additivity) on *P. aeruginosa* and FICI = 2 on *S. aureus*, thus indicating the absence of significant interaction of the peptides with this antibiotic.

Overall, our findings indicate that fibrinogen and thrombin-derived cryptic peptides have additive and, sometimes, synergistic activity thus allowing to speculate that they could also cooperate efficiently in vivo at the wound sites where fibrinogen, fibrin, thrombin and other coagulation factors coexist. Moreover, the strong synergy with colistin and additivity with tobramycin are very interesting from a therapeutic perspective, allowing to hypothesize the feasibility of more effective, combined therapies.

### 2.5. Antibiofilm Activity

**Figure 3:**
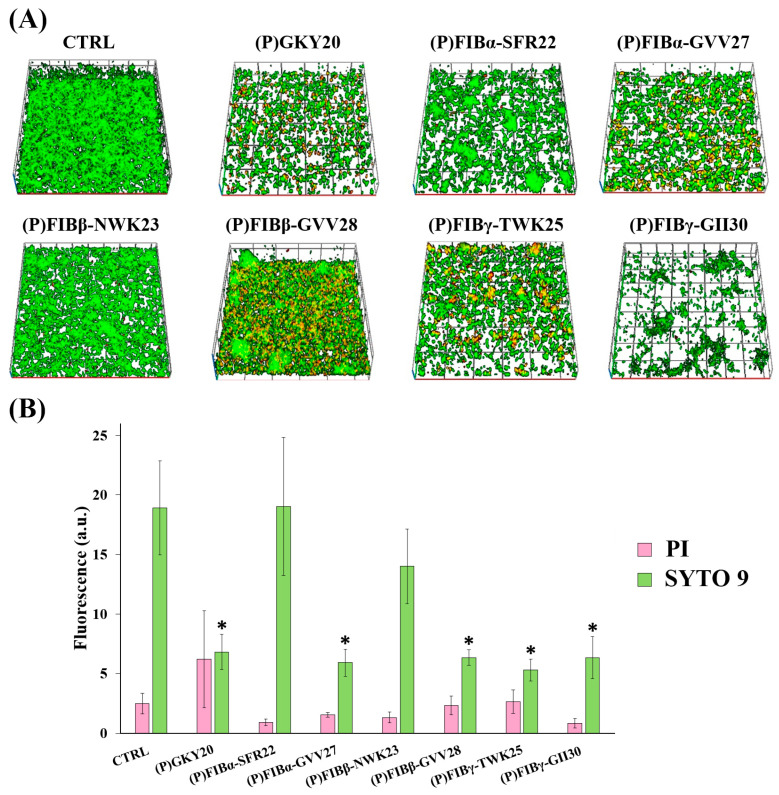
Antibiofilm activity of FIB-derived peptides on *P. aeruginosa* PAO1 biofilms during attachment phase. (**A**) Representative confocal 3D reconstructions (top images) of biofilms stained with the Live/Dead BacLight™ kit (Invitrogen, Waltham, Massachusetts, USA) (green = live bacteria; red = dead bacteria) following peptide treatment (20 µM) during biofilm formation; CTRL: untreated biofilms. (**B**) Quantification of mean fluorescence intensity for SYTO 9 (green bars) and PI (pink bars) (bottom image). Data represent the mean of three independent experiments (biological triplicates), each with at least five internal fields. Error bars are standard errors. Statistical significance was calculated by unpaired two-tailed Student’s *t*-test vs. control *p* < 0.05 (*).

The antibiofilm activity of FIB-derived peptides and of (P)GKY20 was studied on *P. aeruginosa* PAO1 biofilm by laser scanning confocal microscopy (LSCM) using two different assays. In the attachment assay, bacterial cells and peptides were added simultaneously to the chambered cover glass at the same time and biofilms were stained after 4 h, whereas, in the detachment assay, bacterial cells were first allowed to grow for 24 h to form mature biofilm. Peptides were then added, and after a further 24 h of incubation, biofilms were stained to evaluate their antibiofilm effect [73]. FIB-derived peptides exhibited pronounced antibiofilm activity that varied depending on both the peptide and the stage of biofilm development. In the attachment assay, four FIB-derived peptides, namely (P)FIBα-GVV27, (P)FIBβ-GVV28, (P)FIBγ-TWK25 and (P)FIBγ-GII30 significantly reduced fluorescence signal of SYTO 9 (live cells) indicating effective inhibition of biofilm initiation (Figure 3). No significant variation was observed in the case of the propidium iodide (PI) signal. Regarding the remaining two peptides, (P)FIBβ-NWK23 caused a lower, not significant reduction whereas (P)FIBα-SFR22 did not cause any reduction of the SYTO 9 signal. Again, no significant variation of the PI signal was observed.

**Figure 4:**
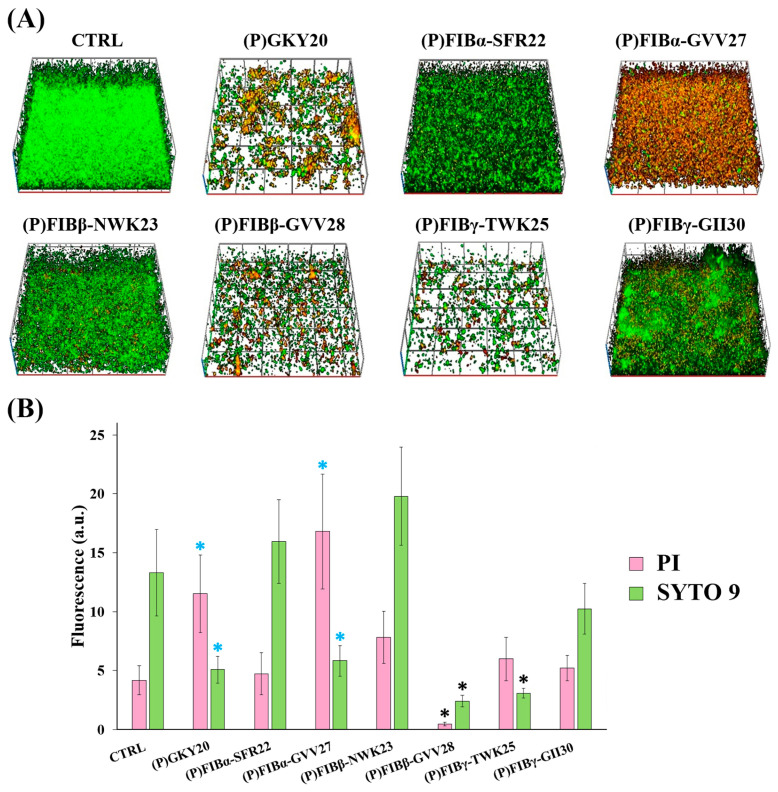
Antibiofilm activity of FIB-derived peptides on *P. aeruginosa* PAO1 biofilms during detachment phase. (**A**) Representative confocal 3D reconstructions (top images) of biofilms stained with the Live/Dead BacLight™ kit (Invitrogen, Waltham, Massachusetts, USA) (green = live bacteria; red = dead bacteria) following peptide treatment (20 µM) on pre-formed biofilms. CTRL: untreated biofilms. (**B**) Quantification of mean fluorescence intensity for SYTO 9 (green bars) and PI (pink bars) (bottom image). Data represent the mean of three independent experiments (biological triplicates), each with at least five internal fields. Error bars are standard errors. Statistical significance was calculated by unpaired two-tailed Student’s *t*-test vs. control. Black asterisks, *p* < 0.05; cyan asterisks, 0.085 > *p* ≥ 0.05.

In the detachment assay reported in Figure 4, only (P)FIBγ-TWK25 and (P)FIBβ-GVV28 demonstrated superior disruptive capacity, significantly reducing the SYTO 9 signal. However, (P)FIBα-GVV27 and (P)GKY20 caused a reduction of the SYTO 9 signal coupled with an increase of the PI signal, with *p* values just above the 0.05 significance threshold (in the range 0.085–0.05). Taken together, these variations suggest that the two peptides have a strong impact on biofilm, increasing the ratio between dead and live cells, as also appreciable in the 3D reconstructions (Figure 4A).

### 2.6. Hemolytic Activity

To assess the safety profile of the FIB-derived peptides, their hemolytic activity was evaluated by measuring the release of hemoglobin from sheep red blood cells after 1-h incubation with increasing peptide concentrations.

**Figure 5:**
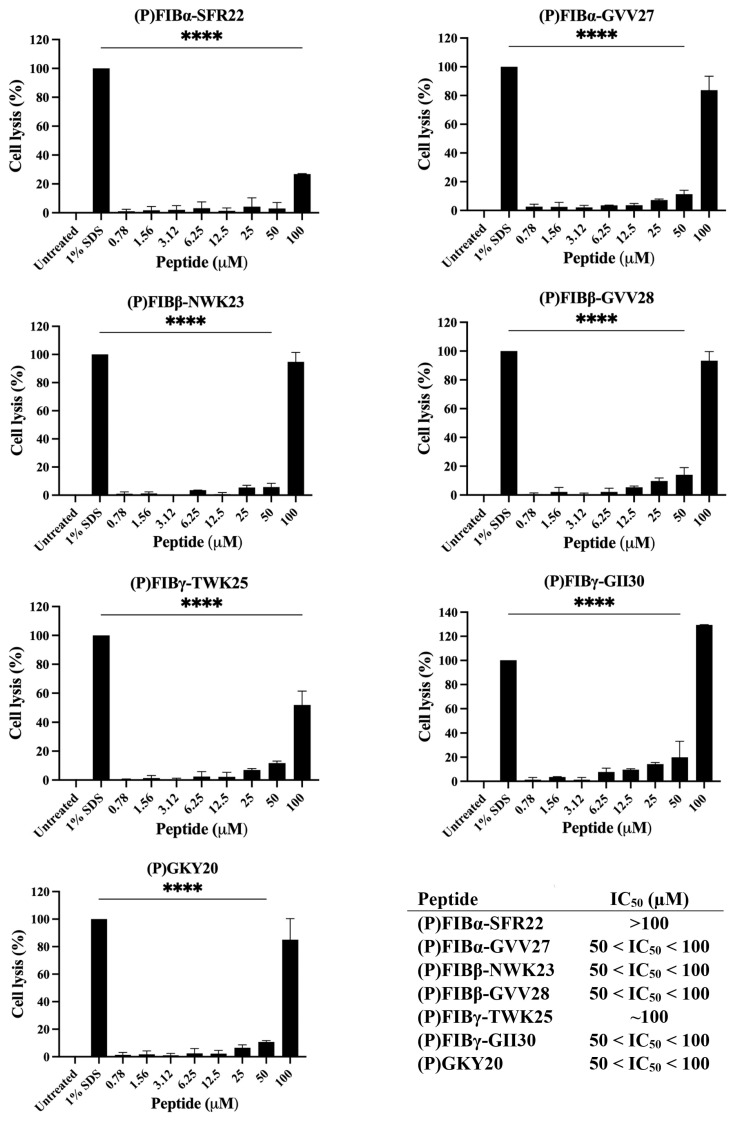
Hemolytic activity of FIB-derived peptides on sheep red blood cells. Graph shows the hemolytic effect on sheep red blood cells of the peptide at increasing concentrations (0.78–100 µM). Cells in SDS 1% (1:1 *v*/*v*) were used as a positive control, while cells in PBS are considered as negative control. IC_50_ values represent the concentration required to induce 50% hemolysis of sheep red blood cells. The experiments were performed in triplicate, and statistical analyses were carried out by using one-way ANOVA test (1% SDS vs. treated samples), (****p < 0.0001).

As shown in Figure 5, all peptides exhibited negligible hemolytic activity at concentrations between 0.78 and 50 µM, with values comparable to the untreated control, and significant hemolysis only at the highest concentration tested (100 µM). However, (P)FIBα-SFR22 and (P)FIBγ-TWK25 showed a limited release of hemoglobin even at 100 µM indicating a more favorable safety profile and the evaluation of IC_50_ further support this interpretation. Importantly, these hemolytic effects were only observed at concentrations much higher than the antimicrobial concentrations required for bacterial killing (see Table 1). This selective toxicity is a key feature of therapeutic peptides and suggests a broad therapeutic window for most FIB-derived peptides tested. These findings are consistent with earlier studies reporting low hemolytic potential for other host defense peptides derived from plasma proteins, such as other thrombin-derived C-terminal peptides and LL-37 analogues [74,75]. Nevertheless, some degree of hemolysis at higher concentrations is not uncommon among amphipathic and cationic peptides, which can interact with zwitterionic lipid membranes of mammalian cells. However, the observed hemolysis remained moderate, especially compared to classical pore-forming toxins or lytic antimicrobial peptides like melittin [76].

### 2.7. Anti-Inflammatory Activity on Murine Macrophages

Several AMPs can attenuate the LPS-induced upregulation of pro-inflammatory mediators and cytokines by LPS-scavenging activity [77]. For this reason, we would verify if FIB-derived peptides could possess such putative anti-inflammatory properties. To collect data on possible anti-inflammatory properties of FIB-derived peptides, the release of nitric oxide (NO) by LPS-treated murine macrophages Raw 264.7 was analysed. Nitric oxide serves multiple functions within biological systems: it acts as a mediator of vasodilation, platelet aggregation, and neurotransmission, and plays a role in regulating the function, survival, and death of various cell types, including many involved in immune response and inflammation [78].

**Figure 6:**
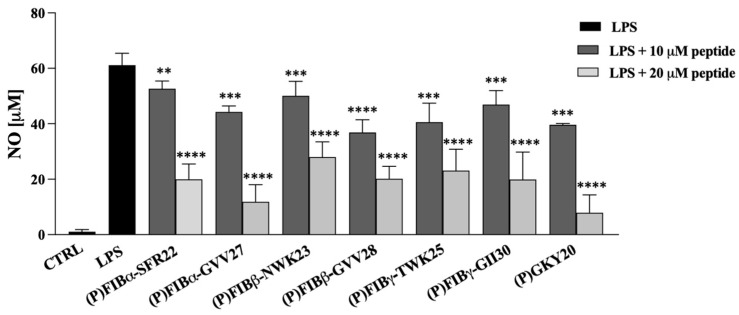
Effects of FIB-derived peptides and the thrombin-derived peptide (P)GKY20 on the release of NO by Raw 264.7 cells stimulated with *P. aeruginosa* 10 LPS. The experiments were performed in triplicate and statistical analyses were carried out by using GraphPad Prism (version 10) and the data presented were the mean values of three experiments ± S.D. The analysis was carried out using Student’s *t*-test (** *p* < 0.01, *** *p* < 0.001 or **** *p* < 0.0001) vs. untreated cells and LPS group. (CTRL: Untreated cells control).

Therefore, Raw 264.7 cells were stimulated by exposure at 100 ng/mL LPS from *P. aeruginosa* 10 and simultaneously treated with 10 or 20 µM of FIB-derived peptides for 24 h (see Methods). (P)GKY20 was used as positive control. After the incubation, the amount of released NO was measured by performing the Griess assay. Once verified that all peptides were biocompatible with Raw 264.7 cells up to 20 µM by using the MTT assay (Supplementary Figure S5) and that they do not induce themself the release of proinflammatory factors in the same range of concentrations (Supplementary Figure S6), we found that all the peptides, when administered to LPS-stimulated cells, effectively decrease LPS-induced release of NO (Figure 6) in a dose-dependent manner.

### 2.8. Binding of Luc-FIBγ-TWK25 to LPS

In order to confirm the hypothesis that anti-inflammatory activity of FIB-derived peptides is due to direct interaction with LPS, we determined K_d_ and binding stoichiometry of FIBγ-TWK25 to two different LPS isolated by *E. coli* 0111:B4 and *P. aeruginosa* 10, respectively. To this aim we prepared peptide Luc-FIBγ-TWK25, labelled at the N-terminus with a luciferin-like, environment-sensitive fluorophore according to an our previously published method [79]. Firstly, peptide (C)FIBγ-TWK25, with a cysteine residue at the N-terminus (Supplementary Figure S1) was obtained by acidic hydrolysis of the fusion protein ONC-DCless-H6-(C)FIBγ-TWK25 that contains the acid labile sequence “Asp-Cys” (whose sensitivity to acidic hydrolysis is just slightly lower than that of the dipeptide “Asp-Pro”). Next, (C)FIBγ-TWK25 was labelled by reaction with 6-hydroxy-2-cyanobenzothiazole (CBT-OH) that undergoes cyclization with the N-terminal cysteine residue, thus generating the luciferin-like fluorophore (Supplementary Figure S7). The emission spectra of Luc-FIBγ-TWK25 after excitation at 400 nm were measured at constant concentration of *E. coli* LPS in the presence of increasing peptide concentrations. The analysis was performed at micellar (40 μg/mL ≈ 4 μM) and sub-micellar (5 μg/mL ≈ 0.5 μM) concentrations of *E. coli* LPS (Critical Micellar Concentration, CMC = 1.3–1.6 µM corresponding to 13–16 µg/mL [80]; MW = ~10 kDa). Fluorescence data (total fluorescence between 450 and 700 nm) were fitted to the model described in [79]. The fitting allowed for the estimation of the K_d_ value and of the number of binding sites. K_d_ values, determined at sub-micellar and micellar LPS, were 61 and 172 nM, respectively, hence in the same order of magnitude even if the peptide: LPS ratio decreased from 4:1 to 1.5:1 (Supplementary Figure S8). This observation may be attributed to the fact that, at concentrations below the CMC, LPS molecules are unassociated, thereby exposing a greater portion of their molecular surface, particularly the fatty acid chains of lipid A which are then free to interact with the peptides, thus causing an increase of the number of peptide molecules that bind to a single LPS molecule. Our findings are very similar to those reported for the thrombin derived peptide GKY20 using an analogous Luc-GKY20 fluorescent peptide and the same LPS [79]. We also studied the interaction of Luc-FIBγ-TWK25 with *P. aeruginosa* 10 LPS at 5 μg/mL, a concentration that can only be presumed to be sub-micellar as CMC and molecular weight of this LPS are not know. Very interestingly both K_d_ value and number of binding sites were similar to those found for LPS from *E. coli*, within the experimental error. Assuming a similar MW for both LPS, it is reasonable to suppose that also the peptide: LPS binding ratio is the same in the studied conditions. This analysis shows that FIBγ-TWK25 binds to LPS with high affinity, supporting the hypothesis that it can serve as an LPS scavenger similarly to GKY20. Obviously, this does not exclude the possibility of further anti-inflammatory effects not mediated by direct peptide/LPS interaction.

### 2.9. Antiviral Activity

**Figure 7:**
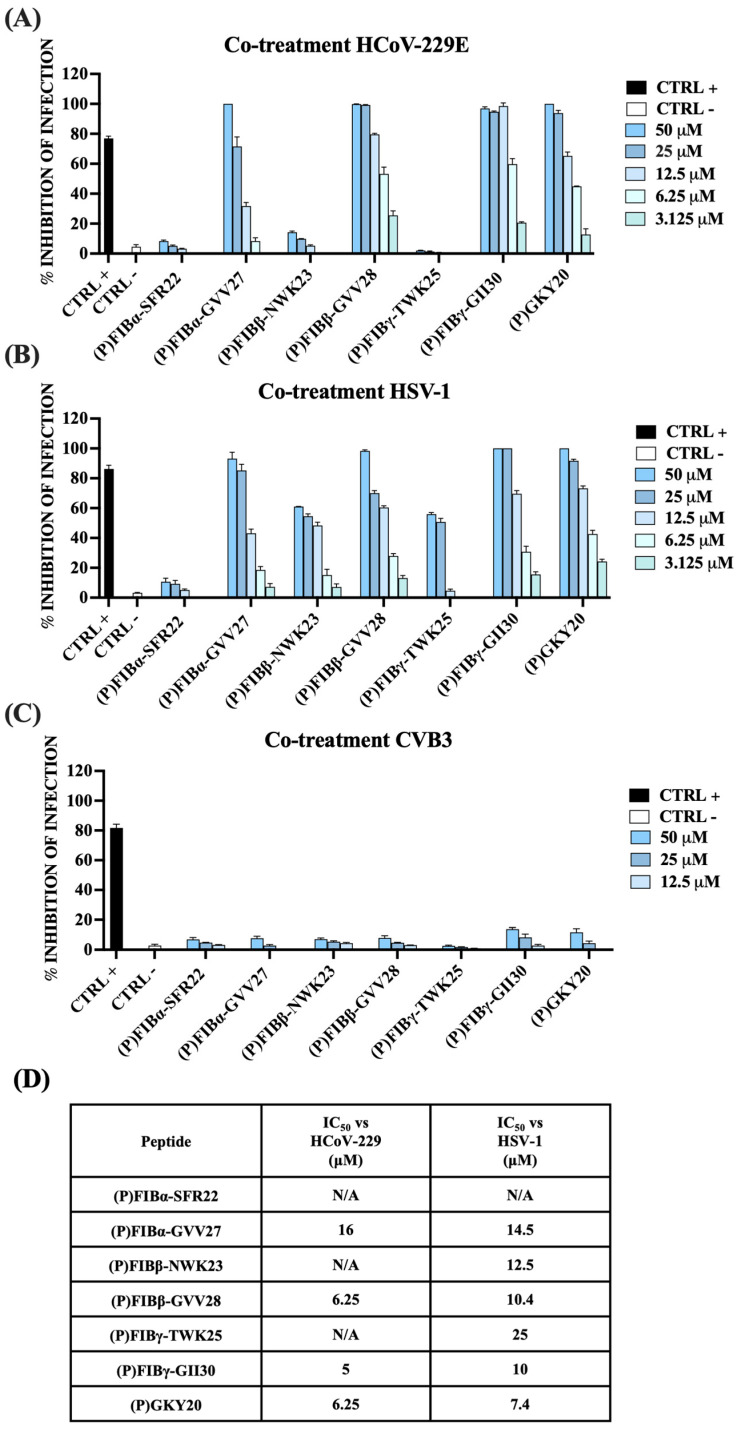
Antiviral activity of human FIB-derived peptides and the thrombin-derived peptide (P)GKY20 in co-treatment assays. Peptides were applied to Vero cell monolayers concurrently with infection by (**A**) HCoV-229E, (**B**) HSV-1, or (**C**) CVB3. After 24 or 48 h, viral plaques were quantified, and the percentage of infection inhibition was calculated as relative to infected untreated controls (CTRL−). Several positive controls (CTRL+) were included for each viral strain: rhamnolipids M15RL (50 μg/mL) for HCoV-229E, melittin (5 μM) for HSV-1, and pleconaril (2 μg/mL) for CVB3. (**D**) IC_50_ values are reported for all peptides against HCoV-229E and HSV-1. N/A: not applicable.

For several AMPs of human origin, such as LL-37 [81,82], it has been reported the ability to interfere with viral infectivity, generally by targeting the envelope of viruses given their amphipathic and cationic nature [83]. On the basis of this premise, we investigated whether selected FIB-derived peptides, in addition to their antibacterial properties, could also inhibit the infection of mammalian Vero cells by enveloped and non-enveloped viruses. Before assessing their antiviral activity, the MTT assay was performed to establish their biocompatibility with Vero cells. Once verified that all peptides were perfectly biocompatible with selected cell line (Supplementary Figure S9) to assess the antiviral potential of the six FIB-derived peptides and of (P)GKY20, we performed co-treatment assays on Vero cells infected with both enveloped and non-enveloped viruses starting from a concentration of 50 μM of each peptide. Specifically, the peptides were tested against the human coronavirus HCoV-229E and herpes simplex virus type 1 (HSV-1), both of which possess a lipid envelope, as well as coxsackievirus B3 (CVB3), a non-enveloped virus lacking a lipid membrane. Initially, a co-treatment assay was conducted in which cells were simultaneously exposed to both the virus and the peptide. As shown in Figure 7 (panel A), (P)FIBα-GVV27, (P)FIBβ-GVV28, (P)FIBγ-GII30, and (P)GKY20 demonstrated strong antiviral activity against HCoV-229E.

Specifically, (P)FIBγ-GII30 proved to be the most effective peptide against HCoV-229E, exhibiting a 50% inhibitory concentration (IC_50_) of 5 μM. This was followed by (P)GKY20 and (P)FIBβ-GVV28 with an IC_50_ of 6.25 μM, and (P)FIBα-GVV27 with an IC_50_ of 16 μM.

Conversely, all peptides demonstrated antiviral activity against HSV-1 in Vero cells, except for (P)FIBα-SFR22, which showed no significant effect (Figure 7B). Similar to the results observed with HCoV-229E, (P)GKY20 and (P)FIBγ-GII30 exhibited the most potent inhibition, with IC_50_ values of 7.4 μM and 10 μM, respectively. (P)FIBβ-GVV28 also showed strong antiviral efficacy, with an IC_50_ of 10.4 μM, while (P)FIBα-GVV27 IC_50_ was 14.5 μM. In contrast to their absence of efficacy against HCoV-229E, peptides (P)FIBγ-TWK25 and (P)FIBβ-NWK23 exhibited modest antiviral activity against HSV-1, with IC_50_ values of 25 μM and 12.5 μM, respectively. A summary of all IC_50_ values is provided in Figure 7D. Intriguingly, no inhibitory effect was observed against CVB3 by all six FIB-derived peptides and (P)GKY20 (Figure 7C). These findings indicate that the peptides exert selective antiviral activity against enveloped viruses, likely through interactions with the viral lipid envelope, and underscore their potential role as components of the innate immune defense. Concerning this, it is worth noting that the most virucidal FIB-derived peptides are the three longer peptides that share the presence of the short hydrophobic tail at the N-terminus (Figure 2). It is plausible to hypothesize that this tail is important for the interaction with the viral membrane, mediating the insertion into and/or stabilizing the binding to the membrane. Considering that viral membranes originate from eukaryotic cell membranes, the fact that the short forms of the FIB-derived peptides (apart from (P)FIBβ-NWK23) are less hemolytic than the longer counterparts is consistent with the hypothesis that the tail plays a role in the interaction with eukaryotic/viral membranes.

**Figure 8:**
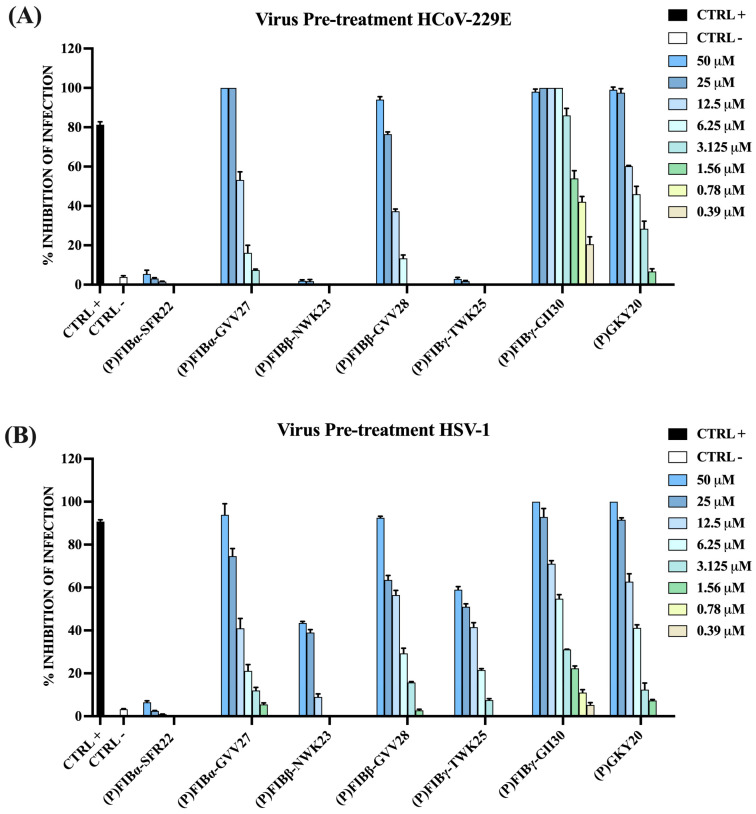
Antiviral activity human FIB-derived peptides and the thrombin-derived peptide (P)GKY20 against HCoV-229E (**A**) and HSV-1 (**B**) in virus pre-treatment assays. Following 24 or 48 h of incubation, viral plaques were counted, and the percentage of infection inhibition was determined in comparison to untreated infected controls (CTRL−). Specific positive controls (CTRL+) were included for each viral strain tested: rhamnolipids M15RL (50 μg/mL) for HCoV-229E, and melittin (5 μM) for HSV-1.

To gain deeper insight into the antiviral mechanisms of the peptides, additional experimental conditions were employed against HCoV-229E and HSV-1. These included: (i) pre-incubation of the virus with peptides prior to infection, (ii) pre-treatment of Vero cells with the peptides before viral exposure, and (iii) treatment with the peptides following viral infection. As shown in Figure 8, all peptides that were active in the co-treatment assays maintained their antiviral effects when pre-incubated with the viruses. In many cases, their efficacy was even enhanced, supporting the hypothesis of a direct interaction between the peptides and viral particles. (P)FIBγ-GII30 exhibited the strongest virucidal effect, with IC_50_ values of 1.3 μM against HCoV-229E and 5.4 μM against HSV-1.

**Figure 9:**
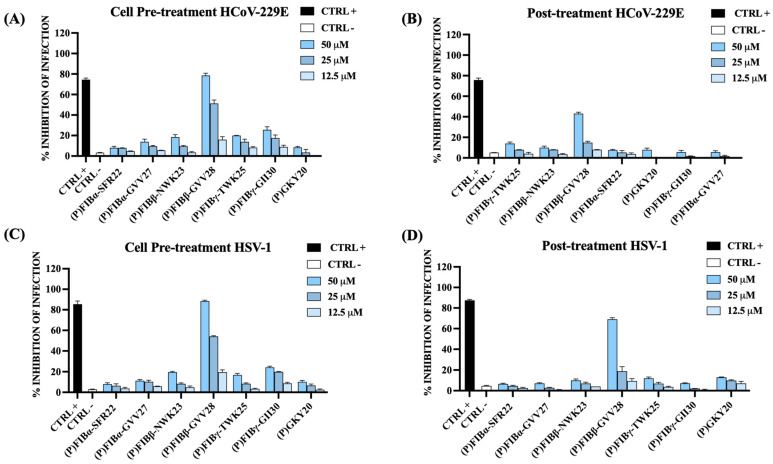
Antiviral activity of human FIB-derived peptides and the thrombin-derived peptide (P)GKY20 against HCoV-229E (**A**,**B**) and HSV-1 (**C**,**D**) in cell pre-treatment and post-treatment assays. Following 24 or 48 h of incubation, viral plaques were counted, and the percentage of infection inhibition was determined in comparison to untreated infected controls (CTRL−). Specific positive controls (CTRL+) were included for each viral strain tested: ivermectin ((**A**) 10 μM), remdesivir ((**B**) 10 μM), dextran sulfate ((**C**) 1 μM), and aciclovir ((**D**) 5 μM).

In contrast, only one peptide, (P)FIBβ-GVV28, showed appreciable antiviral activity in both cell pre-treatment and post-infection treatment conditions, whereas the other peptides were minimally effective (Figure 9). These results indicate that most FIB-derived peptides likely act through direct virucidal or virus-neutralizing mechanisms, while (P)FIBβ-GVV28 may possess additional properties affecting host–virus interactions or early steps of infection. Although further studies are needed, this peptide may engage distinct cellular targets or modulate early virus–host cell processes beyond simple membrane disruption.

## 3. Materials and Methods

### 3.1. Materials

*E. coli* BL21(DE3) was purchased from Novagen (San Diego, CA, USA). Expression vectors pET22b(+)/ONC-DCless-H6-(P)FIBα-GVV27, pET22b(+)/ONC-DCless-H6-(P)FIBα-SFR22, pET22b(+)/ONC-DCless-H6-(P)FIBβ-GVV28, pET22b(+)/ONC-DCless-H6-(P)FIBβ-NWK23, pET22b(+)/ONC-DCless-H6-(P)FIBγ-GII30, pET22b(+)/ONC-DCless-H6-(P)FIBγ-TWK25, pET22b(+)/ONC-DCless-H6-(C)FIBγ-TWK25, coding for the corresponding recombinant fusion proteins, were purchased from GenScript (USA Inc., Piscataway, NJ, USA). Codons were optimized for expression in *E. coli*. Nucleotide and amino acid sequences of peptides are reported in Supplementary Figure S1. Vector pET22b(+)/ONC-DCless-H6-(P)GKY20 was used to produce thrombin-derived cryptic AMP (P)GKY20 [59,84]. Synthetic FIB-derived peptides were obtained using solid-phase peptide synthesis (Fmoc 9-fluorenylmethoxycarbonyl strategy) and purified by HPLC (purity higher than 95%). Synthetic LL-37 (LLGDFFRKSKEKIGKEFKRIVQRIKDFLRNLVPRTES) was from CASLO ApS (Kongens Lyngby, Denmark). Ni Sepharose^TM^ 6 FastFlow was from GE Healthcare (Uppsala, Sweden). Nutrient broth (NB) was purchased from Difco (Detroit, MI, USA). Tryptone and yeast extract were purchased from Condalab (Madrid, Spain). SDS-PAGE was carried out according to Laemmli [85]. All other reagents were from Merck KGaA (Darmstadt, Germany).

### 3.2. In Silico Analysis

The sequences of fibrinogen subunits were analyzed as previously described [33]. Briefly, the “Absolute Score” (AS) of overlapping protein fragments (“windows”) of 12-38 amino acids (“window length”) was calculated with the equation.

AS = (C*^m^*H*^n^*L)/*MaxScore*

where C is the net charge of the fragment, H is the hydrophobicity of the fragment calculated using one of the hydrophobicity scales described in [33], L is the length of the fragment, exponents *m* and *n* are strain dependent weight factors described in [33], and *MaxScore* is a normalization factor depending on L, *m* and *n*. AS values used in this study were calculated using parameters determined for strain *S. aureus* C623 and the “Parker-Arg0” hydrophobicity scale [33]. Contour plots were prepared plotting the AS values as function of the position of the first residue of the window and the window length. AS of a peptide is linearly correlated with its antimicrobial potency, defined as Log (1000/MIC), according to the equation.

AS = *a* [Log (1000/MIC)] + *b*

where *a* and *b* are strain-dependent parameters determined by titration as described previously [33]. The linear relationship is good for AS values up to about 11. For higher AS values, linearity is lost and an increase of AS does not necessarily result in higher potency i.e., lower MIC values.

### 3.3. Expression of the Recombinant Proteins

*E. coli* strain BL21(DE3) was used to express recombinant proteins. Cells, transformed with pET recombinant plasmids (listed in paragraph 3.1 and described in Supplementary Figure S1), were grown in 10 mL of LB medium containing 100 µg/mL ampicillin, at 37 °C up to an absorbance of 2 OD at 600 nm. These cultures were used to inoculate 1 L of LB/ampicillin medium containing 100 µg/mL ampicillin. Cultures were incubated at 37 °C under shaking up to 1.5–2 OD_600 nm_. Expression of recombinant proteins was induced by addition isopropyl β-D-1-thiogalactopyranoside (IPTG) at a final concentration of 0.4 mM. Cells were harvested after 3 h induction by centrifugation at 6000 rpm for 10 min at 4 °C. The bacterial pellet was suspended in 50 mM Tris-HCl, pH 7.4 and sonicated by ultrasonic processor on ice (15 × 1 min cycle; amplitude control set at 100%). The suspension was then centrifuged at 12,000 rpm for 60 min at 4 °C. Soluble and insoluble fractions were analyzed by SDS-PAGE.

### 3.4. Purification of the Recombinant Peptides Procedures

#### 3.4.1. Purification of the Fusion Proteins

Recombinant proteins were purified by immobilized metal ion affinity chromatography (IMAC), using the Ni Sepharose™ 6 Fast Flow resin (Cytiva, Marlborough, MA, USA). Typically, 100 mg to 140 mg of the fusion protein were dissolved in 20 mL of denaturing buffer (5 M guanidine/HCl in 50 mM Tris-HCl, pH 7.4) and incubated on a rotary shaker at 37 °C for 3 h under nitrogen atmosphere. The soluble fraction was collected by centrifugation and incubated with 5-7 mL of Ni Sepharose™ 6 Fast Flow resin equilibrated in denaturing buffer. The resin was shaken at 4 °C for 16 h and then collected by centrifugation. The fusion protein was eluted in 0.1 M sodium acetate buffer, pH 5.0, containing 5 M guanidine/HCl (elution buffer). In the case of the fusion protein ONC-DCless-H6-(C)FIBγ-TWK25, IMAC purification was carried out in the presence of 1 mM Tris(2-carboxyethyl)phosphine hydrochloride (TCEP-HCl), a thiol-free reducing agent, to obtain the protein in the reduced form. The eluate was extensively dialyzed against 0.1 M acetic acid at 4 °C. Samples were stored at −80 °C under nitrogen atmosphere. The concentration of the purified fusion proteins was determined by spectrophotometric analysis, employing extinction coefficients calculated using the ProtParam tool (Supplementary Table S1).

#### 3.4.2. Cleavage of Asp-Pro and Asp-Cys Peptide Bonds

Cleavage of peptides from the fusion proteins was performed in 0.1 M acetic acid containing 18 mM HCl (pH 2.0) at 60 °C for 24 h in a water bath under nitrogen atmosphere in the presence of 1 mM TCEP-HCl as scavenger of reactive oxygen species. Cleavage percentage was estimated by SDS-PAGE analyses RP-HPLC.

#### 3.4.3. Purification of the Recombinant Peptides

Peptides (P)FIBα-GVV27, (P)FIBα-SFR22, (P)FIBβ-NWK23 and (P)FIBγ-TWK25 were separated from the carrier and the uncleaved fusion proteins as previously described [59]. The hydrolysis mixture was neutralized to pH 7–7.2 by adding 1 M NH_3_ and incubated for 1 h at 25 °C under nitrogen atmosphere in a water bath. The uncleaved fusion protein and the carrier (insoluble at pH 7) were separated from the peptide (soluble at pH 7) by centrifugation (1 h at 12,000 rpm at 4 °C). Supernatant and insoluble fraction were analyzed by SDS-PAGE. Concentration of the peptide into the soluble fraction was determined by spectrophotometric analyses using extinction coefficients (Supplementary Table S1) calculated by the ProtParam tool [86]. After the selective precipitation step, purification of the peptide was performed by RP-HPLC. The linear gradients used are listed in Supplementary Table S2. Purification of (P)FIBβ-GVV28 and (P)FIBγ-GII30 was performed directly by RP-HPLC, omitting the selective precipitation step. Purified peptides were lyophilized and dissolved in deionized water. Samples were stored at −80 °C under nitrogen atmosphere. Purity of peptides was evaluated by RP-HPLC on Europa Protein 300 C18 column (5 μm, 25 × 1) using the linear gradients listed in Supplementary Table S2. The identity of the peptides was confirmed by mass spectrometry [87].

#### 3.4.4. RP-HPLC Analyses

RP-HPLC were performed on a Jasco LC-4000 system equipped with PU-4086 semipreparative pumps and an MD-4010 photo diode array detector (JASCO Corporation, Hachiōji, Tokyo, Japan). The column was a C18 (25 × 1 cm, 5 μm particle size) Europa Protein 300 Å (pore size) from Teknokroma (Barcelona, Spain). Gradients used to perform analytical chromatography and purification procedures are reported in Supplementary Table S2.

### 3.5. Labeling of (C)FIBγ-TWK25 Peptide and Purification of Luc-FIBγ-TWK25 Peptide

(C)FIBγ-TWK25, obtained by chemical hydrolyses of ONC-DCless-H6-(C)FIBγ-TWK25 protein, was labeled by 6-hydroxy-2-cyanobenzothiazole (CBT-OH), a site-specific N-terminal cysteine modification reagent. Labeling was performed following the procedure previously described [70,84], with minor modifications. Briefly, labeling reaction was performed directly on hydrolysis mixture in 15 mM sodium phosphate buffer (NaP), pH 7.4, containing 3 M guanidine-HCl, 2 mM TCEP and 0.5 mM CBT-OH (100 mM stock solution in dimethylformamide), at final peptide concentration of about 60 µM. To avoid carrier precipitation at pH 7.4, guanidine-HCl was added to the hydrolysis mixture at pH 2.0, before increasing the pH to 7.4 by addition of diluted NH_3_. The sample was incubated at 25 °C for 2 h in the dark under nitrogen atmosphere. Control mixture without peptide was also prepared. The modification reaction was monitored by RP-HPLC on Europa Protein 300 C18 column using linear gradient 3 (Supplementary Table S2). Excess CBT-OH was quenched by adding L-cysteine (Cys:CBT-OH molar excess = 10:1; 1 h at 37 °C in the dark). Reaction was stopped by adding 1% TFA. The peptide was purified by RP-HPLC on Europa Protein 300 C18 column using gradient 1 (Supplementary Table S2). Purified peptide was lyophilized and dissolved in deionized water. Its concentration was determined using the luciferin molar extinction coefficients reported in literature (ε_322nm_ = 18,340 M^−1^ cm^−1^) [88]. Peptide concentration was also confirmed by BCA assay (following the instructions of manufacturer). Purity of the peptide was assessed by RP-HPLC on C18 column (gradient 3, see Supplementary Table S2).

### 3.6. Antibacterial Assays: MIC, MBC and FIC Index

MIC values were determined on Gram-positive and Gram-negative bacteria by the broth microdilution method for antimicrobial peptides previously described [33,89] with minor modifications.

Assays were carried out in Nutrient Broth 0.5× (Difco, Detroit, MI, USA) using sterile 96-well polypropylene microtiter plates (cat. 3879, Costar Corp., Cambridge, MA, USA). Bacterial strains were grown in Luria-Bertani (LB) medium overnight at 37 °C and then diluted in Nutrient Broth at a final concentration of ~5 × 10^5^ CFU/mL per well. Twofold serial dilutions of peptides were carried out in the test wells to obtain concentrations ranging from 50 μM to 0.05 μM. Plates were incubated overnight at 37 °C.

MIC value was defined as the lowest concentration at which growth was inhibited. Three independent experiments were performed for each MIC value. The antibiotics polymyxin B and vancomycin (Merck KGaA, Darmstadt, Germany) were tested as control (twofold serial dilutions starting from 64 µg/mL concentration).

MBC values were determined on the overnight cultures used to perform the MIC tests by subculturing on LB agar plates. The MBC value was defined as the lowest concentration of antibacterial agent that kills  ≥ 99.9% of bacterial cells

Synergy between AMPs and AMP/antibiotic combinations was assessed by the so-called checkerboard assay, a broth microdilution assay based on a two-dimensional array of serial dilutions of tested compounds [72]. Experiments were carried out in 96-well plate on *P. aeruginosa* PAO1 (Gram-negative) and *S. aureus* ATCC 6538P (Gram-positive). Plates were incubated 16 h at 37 °C. FIC index was calculated as follows: FIC_A_ + FIC_B_, where FIC_A_ = MIC of drug A in combination/MIC of drug A alone, and FIC_B_ = MIC of drug B in combination/MIC of drug B alone.

### 3.7. Confocal Microscopy-Based Biofilm Analysis

To assess the antibiofilm activity of FIB-derived peptides, *P. aeruginosa* PAO1 biofilms were grown in glass-bottomed multiwell chambers (Nunc™ Lab-Tek™ Chambered Coverglass, Thermo Fisher Scientific, Waltham, MA, USA) and treated with peptides at a final concentration of 20 µM either during biofilm formation (attachment assay) or on mature biofilms (detachment assay). Biofilms were stained using the Live/Dead BacLight™ Bacterial Viability Kit (Invitrogen, Waltham, MA, USA), which uses SYTO 9 (green fluorescence, total cells) and propidium iodide (PI) (red fluorescence, dead/damaged cells) [38,90]. Imaging was performed with a Zeiss LSM 600 confocal laser scanning microscope (Carl Zeiss AG, Oberkochen, Germany), and 3D biofilm structures were reconstructed using dedicated Zeiss software (Zen blue edition software version 2.3 lite). Quantitative analysis of mean fluorescence intensity and biofilm thickness was performed on at least five independent fields per sample.

### 3.8. Hemolysis Assay

The release of hemoglobin from Sheep Red Blood Cells (10% washed pooled cells, Rockland Immunochemicals, Inc., 321 Jones Boulevard, Limerick, UK) was used as a measure for the hemolytic activity of FIB-derived peptides and for the control peptide (P)GKY20 by using the protocol previously optimized [39,40,91]. Briefly, Sheep Red Blood Cells were diluted in PBS 1× to reach a final concentration of 2%. Aliquots of diluted erythrocytes (100 μL) were added to each peptide solution (0–100 μM; 100 μL) in 96-well microtiter plates, and the mixture was incubated for 30 min under shaking (3000 rpm) at 37 °C. Following the incubation, a 3 min centrifugation at 4 °C was performed, and 50 µL of the supernatant from each sample were transferred to a 96 well plate. Absorbance values were determined at 405 nm by using an automatic multiple reader (Benchmark Plus Microplate Spectrophotometer, Bio-Rad, Hercules, CA, USA). Hemolysis percentage was calculated by comparing samples to controls containing PBS (negative control) and 1% SDS (*v*/*v*) in PBS solution (positive control, inducing complete lysis).

$$\mathrm{Hemolysis}\left(\%\right)=\frac{\left(Abs405\;nm\;peptide-Abs405\;nm\;negative\;control\right)}{\left(Abs405\;nm\;positive\;control-Abs405\;nm\;negative\;control\right)}\times 100.$$

### 3.9. MTT Assay

Cytotoxic effects of the peptides on Raw 264.7 cells were determined by performing the 3-(4,5-dimethylthiazol-2-yl)-2,5 diphenyltetrazolium bromide reduction inhibition assay (MTT assay), designed to be used for the spectrophotometric quantification of cell proliferation. Briefly, 2 × 10^4^ cells were seeded into a 96-well plate and incubated at 37 °C in the presence of 5% CO_2_. The medium was subsequently replaced with 100 µL of fresh medium containing the peptide solution at final concentrations ranging from 0.3 to 20 µM/well. After 6 and 24 h of incubation at 37 °C, the peptide-containing medium was removed, and 100 µL of tetrazolium MTT diluted at 0.5 mg/mL in Dulbecco’s modified Eagle’s medium (DMEM) purchased from Lonza (Basel, Switzerland) without red phenol was added. Following a 4-h incubation at 37 °C, the insoluble formazan salts were dissolved in 0.04 M HCl in anhydrous isopropanol and quantified by measuring absorbance at 570 nm using an automated plate reader spectrophotometer (Synergy™ H4, BioTek, Winooski, VT, USA). Cell viability was expressed as the mean percentage relative to the control. Each analysis was performed at least three times [92,93].

### 3.10. Immune-Modulatory Activity Analysis

The ability of FIB-derived peptides to modulate nitric oxide release in RAW 264.7 cells was measured via a Griess assay [94,95,96]. Cells (2 × 10^4^ cells/well) were seeded into 96-well microtiter plates. The next day, the culture medium was discarded and replaced with a fresh medium containing either (i) a mixture of each FIB-derived peptide and 100 ng/mL LPS from *P. aeruginosa* 10 (co-incubation), (ii) only each FIB-derived peptide (20 μM) or (iii) only 100 ng/mL LPS from *P. aeruginosa 10*. Cell supernatants were collected after 24 h of incubation at 37 °C and 5% CO_2_. Nitrite concentrations were measured using a colorimetric assay with the Griess Reagent Kit for nitrite quantitation (Invitrogen™, Waltham, MA, USA). Briefly, cell culture supernatants were combined with equal volumes of N-(1-naphthyl) ethylenediamine (Component A) and sulfanilic acid (Component B) to form the Griess Reagent, followed by a 30-min incubation at room temperature. The absorbance was measured at 548 nm using a 96-well microplate reader (SynergyTM H4, BioTek, Winooski, VT, USA).

### 3.11. Interaction Between Luc-FIBγ-TWK25 Peptide and LPS: K_d_ and Binding Stoichiometry

Fluorescent spectra were recorded in 10 mM NaP buffer, pH 7.4, at 25 °C in the presence of *P. aeruginosa 10* LPS (5 μg/mL) and *E. coli* 0111:B4 LPS (5 μg/mL and 40 μg/mL ≈ 4 μM). Luc-FIBγ-TWK25 (0.25–18 μM) was added to mixtures and incubated 15 min before fluorescent emission at 539 nm was recorded by excitation at 400 nm (phenolate form). The assays to determine K_d_ and binding stoichiometry of Luc-peptide toward LPS were carried out in 96-well polystyrene microtiter plates containing 100 μL of peptide/LPS mixtures, using a Synergy^TM^ H4 microplate reader (BioTek Instruments Inc., Winooski, VT, USA). Emission spectra were recorded in the range 450 nm–700 nm. The experimental data were fitted to the model described in [79]. Areas below emission spectra were plotted toward Luc-FIBγ-TWK25 concentration and K_d_ value was obtained by GraphPad prism software (version 6).

### 3.12. Antiviral Activity Methods

#### 3.12.1. Cells and Viruses

Vero cells (ATCC CRL-1587, Manassas, VA, USA), obtained from the American Type Culture Collection (Manassas, VA, USA), were maintained in Dulbecco’s Modified Eagle Medium (DMEM; Microtech, Naples, Italy) enriched with 4.5 g/L glucose, 10% fetal bovine serum (FBS; Microtech), 100 IU/mL penicillin and 100 μg/mL streptomycin (Himedia, Naples, Italy). These cells served for the cultivation of human coronavirus 229E (HCoV-229E, ATCC VR-740), herpes simplex virus type 1 (HSV-1, strain SC16), and coxsackievirus B3 (CVB3, strain Nancy, ATCC VR-30), following procedures previously outlined [97,98].

#### 3.12.2. Cytotoxicity on Vero Cells

To assess peptide-induced cytotoxicity, Vero cells were seeded into 96-well plates at a density of 2 × 10^3^ cells per well. The cells subsequently received treatments with a range of peptide concentrations (from 12.5 μM to 50 μM) over 24 h. After incubation, the medium was removed and replaced with an MTT solution (5 mg/mL). Following a 3-h incubation at 37 °C, the generated formazan crystals were dissolved in pure DMSO. Cell viability was evaluated by measuring the absorbance at 540 nm using a spectrophotometer, and it was calculated as a percentage in comparison to the untreated control group (CTRL+).

#### 3.12.3. Antiviral Activity Assay

The antiviral activity of the peptides was investigated using four different treatment strategies: (i) co-treatment, (ii) virus pre-treatment, (iii) cell pre-treatment, and (iv) post-treatment [9,10,11,39]. During co-treatment, Vero cells were simultaneously exposed to the peptide (at non-toxic concentrations) and virus (MOI of 0.01 pfu/mL) for 1 h at 37 °C. In the virus pre-treatment setup, the virus (1 × 10^4^ pfu/mL) was first incubated with the peptide at 37 °C before being added to the cells for an additional hour. For the cell pre-treatment condition, peptides were added to the cells 1 h before infection. In contrast, the post-treatment assay involved infecting the cells first, followed by a 1-h peptide treatment at 37 °C. After each experimental condition, citrate buffer (pH 3) was used to inactivate any remaining virus, and the cells were subsequently filled with 5% carboxymethylcellulose (Sigma-Aldrich, Darmstadt, Germany) in complete medium. Once the cytopathic effect was present, the cell monolayers were fixed with 4% formaldehyde and stained using 0.5% crystal violet. Viral inhibition was quantified by plaque counting, and the percentage of inhibition was determined by comparing plaque numbers in treated samples to those observed in untreated virus-infected controls (CTRL−).

### 3.13. Statistical Analyses

Each experiment was independently repeated three times (biological triplicates) with at least five internal acquisitions for each replicate. Statistical significance between untreated and peptide-treated groups was evaluated using a two-tailed unpaired Student’s *t*-test and one-way ANOVA test, with *p* < 0.05 considered significant. Results are presented as mean ± standard deviation (SD).

## 4. Conclusions

The in-depth characterization of the FIB-derived peptides confirms that the C-termini of the three subunits of human fibrinogen host cathelicidin-like regions that are a potential source of bioactive cryptides. These regions add to the other biologically active sequences already known to be present in the extensions of FIBγ isoforms, thus indicating that the C-terminus of fibrinogen is a sort of hub involved both in clotting regulation and direct antimicrobial defense. All the six investigated cryptides have shown wide spectrum bactericidal activity as expected from membrane-targeting cathelicidin-like AMPs, moreover, at least one of the six, namely FIBγ-TWK25, was as active as (P)GKY20, the well-known cryptic AMP derived from the C-terminus of human thrombin. Very interestingly, the FIB-derived peptides always showed additive and, in some cases, synergistic interactions among themselves and with (P)GKY20 as well as with antibiotics belonging to two classes often used in the clinical practice against ESKAPEE pathogens, i.e., polymyxins and aminoglycosides. From a physiological perspective, this finding supports the hypothesis that, in the frame of innate immunity, a net of AMPs and cryptic AMPs could cooperate to provide direct antibacterial defense. On the other hand, from a therapeutic point of view, this suggests that the use of AMPs mixtures could probably be a much more effective strategy against MDR pathogens. The fact that each of these peptides has its own peculiar combination of antibiofilm, LPS-neutralization and virucidal activity makes even more attractive the pharmacological exploitation of the single peptides and of their mixtures.

The co-existence of antibacterial activity and virucidal activity toward enveloped virus is not uncommon and not particularly surprising among membrane-targeting AMP, however, two aspects of the FIB-derived peptides make them particularly intriguing. First, the fact that only the longer versions of the peptides are active indicates that the hydrophobic tail is a structural feature essential for full virucidal activity and provides insights into a future rational designing of antiviral peptides. Second, the fact that the long form of the peptide derived from FIBβ also possesses the ability to inhibit infection when administered before or after the addition of the virus indicates the existence of specific mechanisms unrelated to viral envelope damage. Since these abilities are not present in peptides derived from FIBα and γ, despite their homology and strong sequence similarity, the system consisting of the three long peptides represents an excellent way to investigate the molecular determinants of these intriguing activities.

Finally, we emphasize that all peptides were efficiently produced in recombinant form thanks to the ONC carrier. We are currently exploring the possibility of expressing ONC fusion proteins containing three or more AMPs, aiming to obtain equimolar mixtures of peptides through the cleavage of a single recombinant protein. This strategy would make feasible the pharmacological exploitation of complex AMP mixtures.

## Figures and Tables

**Table 1:** Antimicrobial activity (MIC values) of recombinant and synthetic FIB-derived peptides.

	MIC (µM)									
	Gram-Negative								Gram-Positive	
	*E. coli* ^a^	*K. pne.* ^b^	*A. bau.* ^c^	*S. ent.* ^d^	*S. typ.* ^e^	PAO1 ^f^	PA14 ^g^	RP73 ^h^	*S. aur.* ^i^	*E. fae.* ^l^
**FIBα-GVV27**	12.5	25	6.25	12.5	6.25	6.25	6.25	3.12	6.25	6.25
**(P)FIBα-GVV27**	12.5	50	6.25	12.5	6.25	6.25	6.25	3.12	6.25	6.25
**FIBα-SFR22**	25	>50	25	50	50	12.5	12.5	12.5	12.5	6.25
**(P)FIBα-SFR22**	25	>50	25	>50	25	12.5	12.5	6.25	12.5	6.25
**FIBβ-GVV28**	6.25	25	6.25	12.5	12.5	6.25	6.25	6.25	3.12	6.25
**(P)FIBβ-GVV28**	6.25	25	6.25	12.5	12.5	6.25	6.25	6.25	3.12	6.25
**FIBβ-NWK23**	6.25	25	6.25	12.5	12.5	6.25	3.12	6.25	3.12	6.25
**(P)FIBβ-NWK23**	6.25	25	6.25	12.5	6.25	6.25	3.12	3.12	3.12	6.25
**FIBγ-GII30**	6.25	12.5	6.25	12.5	6.25	6.25	6.25	3.12	6.25	6.25
**(P)FIBγ-GII30**	12.5	25	6.25	12.5	6.25	12.5	6.25	3.12	6.25	6.25
**FIBγ-TWK25**	3.12	6.25	3.12	6.25	3.12	3.12	3.12	3.12	1.56	6.25
**(P)FIBγ-TWK25**	3.12	6.25	3.12	3.12	3.12	3.12	3.12	3.12	1.56	3.12
**(P)GKY20**	6.25	6.25	3.12	6.25	3.12	3.12	6.25	1.56	1.56	1.56
**ANTIBIOTICS**	**MIC (µg/mL)**									
**Vancomycin**									0.5	2
**Polymyxin B**	0.5	0.5	0.5	0.5	0.12	0.25	0.25	0.12		
	**Grey: >50**			**4 < MIC ≤ 9**						
**Color code**	**18 < MIC ≤ 50**			**1.6 < MIC ≤ 4**						
	**9 < MIC ≤ 18**			**≤1.6**						

Data were obtained from a minimum of three independent experiments. (P)GKY20, vancomycin and polymyxin B were used as controls. **^a^ *E. coli*:** *Escherichia coli* ATCC 25922; **^b^ *K. pne.*:** *Klebsiella pneumonia* ATCC 700603; **^c^ A. *bau.*:** *Acinetobacter baumanii* ATCC 17878; **^d^ *S. ent.*:** *Salmonella enteriditis* 706 RIVM; **^e^ *S. typ.*:** *Salmonella typhimurium* ATCC 14028; **^f^ *PAO1*:** *Pseudomonas aeruginosa* PAO1; **^g^ *PA14*:** *Pseudomonas aeruginosa* PA14; **^h^ *RP73*:** *Pseudomonas aeruginosa* RP73 (clinical isolate); **^i^ *S. aur*:** *Staphylococcus aureus* ATCC 6538P; **^l^ *E. fae.*:** *Enterococcus faecalis* ATCC 29212.

**Table 2:** Comparison between MIC and MBC values of the peptides.

Peptide/Antibiotic	MIC ^a^ (μM)	MBC ^b^ (μM)	MBC/MIC	MIC ^a^ (μg/mL)	MBC ^b^ (μg/mL)
	***Pseudomonas aeruginosa* PAO1**				
**(P)FIBα-GVV27**	6.25	6.25	1		
**(P)FIBα-SFR22**	12.5	12.5	1		
**(P)FIBβ-GVV28**	6.25	6.25	1		
**(P)FIBβ-NWK23**	6.25	6.25	1		
**(P)FIBγ-GII30**	12.5	12.5	1		
**(P)FIBγ-TWK25**	3.12	3.12	1		
**(P)GKY20**	3.12	3.12	1		
**LL37**	1.56	1.56	1		
**Colistin**			1	0.5	0.5
**Tobramycin**			2	0.0312	0.0625
**Ciprofloxacin**			2	0.125	0.25
	***Staphylococcus aureus* ATCC 6538P**				
**(P)FIBα-GVV27**	6.25	6.25	1		
**(P)FIBα-SFR22**	12.5	12.5	1		
**(P)FIBβ-GVV28**	3.12	3.12	1		
**(P)FIBβ-NWK23**	3.12	3.12	1		
**(P)FIBγ-GII30**	6.25	6.25	1		
**(P)FIBγ-TWK25**	1.56	1.56	1		
**(P)GKY20**	1.56	1.56	1		
**LL37**	1.56	1.56	1		
**Vancomycin**			1	0.5	0.5
**Tobramycin**			2	0.125	0.25
**Ciprofloxacin**			2	0.125	0.25

Data were obtained from a minimum of three independent experiments. ^a^ MIC: Minimum Inhibitory Concentration. ^b^ MBC: Minimum Bactericidal Concentration.

**Table 3:** Fractional Inhibitory Concentration Index (FICI) analysis.

			ΣFICI ^a^						
	Peptides		(P)FIBα-GVV27	(P)FIBα-SFR22	(P)FIBβ-GVV28	(P)FIBβ-NWK23	(P)FIBγ-GII30	(P)FIBγ-TWK25	(P)GKY20
***Pseudomonas aeruginosa* PAO1**	**peptides**	**(P)FIBα-GVV27**	na ^b^						
		**(P)FIBα-SFR22**	-	na ^b^					
		**(P)FIBβ-GVV28**	1	-	na ^b^				
		**(P)FIBβ-NWK23**	-	**0.56**	-	na ^b^			
		**(P)FIBγ-GII30**	0.62	-	**0.56**	-	na ^b^		
		**(P)FIBγ-TWK25**	-	1	-	0.75	-	na ^b^	
		**(P)GKY20**	0.750	-	0.625	-	**0.50**	**0.56**	na ^b^
	**antibiotics**								
		**Colistin**	**0.31**	**0.25**	**0.19**	**0.31**	**0.16**	**0.19**	**0.19**
		**Tobramycin**	0.62	0.75	**0.53**	**0.52**	**0.50**	0.62	0.75
		**Ciprofloxacin**	1	1	1	0.75	0.75	1	2
								
***Staphylococcus aureus* ATCC 6538P**	**peptides**	**(P)FIBα-GVV27**	na ^b^						
		**(P)FIBα-SFR22**	-	na ^b^					
		**(P)FIBβ-GVV28**	2	-	na ^b^				
		**(P)FIBβ-NWK23**	-	0.75	-	na ^b^			
		**(P)FIBγ-GII30**	0.75	-	0.62	-	na ^b^		
		**(P)FIBγ-TWK25**	-	1	-	0.62	-	na ^b^	
		**(P)GKY20**	0.62	-	0.75	-	**0.5**	**0.56**	na ^b^
	**antibiotics**								
		**Tobramycin**	0.75	0.62	1	0.75	0.75	0.75	**0.5**
		**Ciprofloxacin**	2	2	2	2	2	2	2
							

^a^ FICI: Fractional Inhibitory Concentration Index. Numbers in bold on yellow background indicate synergistic effects. Regular numbers on green background indicate additive interactions according to EUCAST model [70]. Numbers in bold on green background indicate values above but close to the 0.5 threshold. Numbers on gray background indicate no interaction. ^b^ Not applicable. (-) Not measured.

## Data Availability

All data generated or analyzed during this study are included in this published article (and its Supplementary Files).

## Supplementary Materials

The following supporting information can be downloaded at: https://www.mdpi.com/article/10.3390/ijms26188914/s1.

## Abbreviations

The following abbreviations are used in this manuscript:

FIB	Fibrinogen
CAMPs	Cationic antimicrobial peptides
LPS	Lipopolysaccharide
AMPs	Antimicrobial peptides
MIC	Minimum Inhibitory Concentration
AS	Absolute score
ONC	Onconase
LB	Luria- Bertani
IMAC	Immobilized metal ion affinity chromatography
RP-HPLC	Reverse Phase High-Performance Liquid Chromatography
MBC	Minimum Bactericidal Concentration
FICI	Fractional Inhibitory Concentration Indexes
LSCM	Laser scanning confocal microscopy
PI	Propidium iodide
CTRL	Control
SD	Standard deviation
EPS	Extracellular polymeric substance
IC_50_	Inhibitory Concentration 50
MTT	3-[4,5-dimethylthiazol-2-yl]-2,5 diphenyl tetrazolium bromide
SDS	Sodium Dodecyl Sulfate
PBS	Phosphate-buffered saline
NO	Nitric oxide
Luc	Luciferin
CBT-OH	6-hydroxy-2-cyanobenzothiazole
CMC	Critical micellar concentration
MW	Molecular weight
HCoV-229E	Human coronavirus
HSV-1	Herpes simplex virus type 1
CVB3	Coxsackievirus B3
SDS-PAGE	Dodecyl Sulphate PolyAcrylamide Gel Electrophoresis
OD	Optical density
IPTG	Isopropyl β-D-1-thiogalactopyranoside
Tris-HCl	Tris(hydroxymethyl)aminomethane hydrochloride
RPM	Revolutions for minute
Guanidine/HCl	Guanidine hydrochloride
HCl	Hydrogen chloride
TFA	Trifluoracetic acid
TCEP	Tris(2-carboxyethyl)phosphine
NaP	Sodium phosphate
BCA	Bicinchoninic acid
DMEM	Dulbecco’s Modified Eagle Medium
FBS	Fetal bovine serum
DMSO	Dimethyl sulfoxide
MOI	Multiplicity of infection
