# Supplementary material for: Exploring the Antimicrobial and Antiviral Properties of Cryptic Peptides from Human Fibrinogen
Source: Int J Mol Sci. 2025 Sep 12;26(18):8914. doi: 10.3390/ijms26188914 (PMC12469503; doi:10.3390/ijms26188914)
Supplement: Supplementary file 1 [file ijms-26-08914-s001.zip › IJMS - Supplementary material - Draft Fibrinogen-derived peptides .pdf]

Supplementary Materials

Exploring the antimicrobial and antiviral properties of cryptic peptides from human fibrinogen

Andrea Bosso[1, †, *], Antonio Masino[1,2,3, †], Ilaria Di Nardo[1], Carla Zannella[4], Rosa Gaglione[5], Ida Palumbo[1], Rosanna Culurciello[1], Anna De Filippis[4], Marcelo D. T. Torres[6,7,8,9], Cesar de la Fuente-Nunez[6,7,8,9], Massimiliano Galdiero[4], Angela Arciello[5], Antimo di Maro[10], Elio Pizzo[1,11], Valeria Cafaro[1, ‡], Eugenio Notomista[1, ‡]

[1] Department of Biology, University of Naples Federico II, 80126 Naples, Italy

[2] Department of Translational Medical Science, University of Naples Federico II, Naples, Italy

[3]ImmunoNutritionLab at CEINGE Advanced Biotechnologies, University of Naples Federico II, Naples, Italy [4]Department of Experimental Medicine, University of Campania "Luigi Vanvitelli", 80138 Napoli, Italy

[5] Department of Chemical Sciences, University of Naples Federico II, 80126 Naples, Italy

[6] Machine Biology Group, Departments of Psychiatry and Microbiology, Institute for Biomedical Informatics, Institute for Translational Medicine and Therapeutics, Perelman School of Medicine, University of Pennsylvania, Philadelphia, PA 19104, USA

[7] Departments of Bioengineering and Chemical and Biomolecular Engineering, School of Engineering and Applied Science, University of Pennsylvania, Philadelphia, PA 19104, USA

[8] Department of Chemistry, School of Arts and Sciences, University of Pennsylvania, Philadelphia, PA 19104, USA

[9] Penn Institute for Computational Science, University of Pennsylvania, Philadelphia, PA 19104, USA

[10] Department of Environmental, Biological and Pharmaceutical Sciences and Technologies (DiSTABiF), University of Campania 'Luigi Vanvitelli', Via Vivaldi 43, 81100 Caserta, Italy

[11] Centro Servizi Metrologici e Tecnologici Avanzati (CeSMA), Complesso Universitario di Monte Sant'Angelo, Via Cinthia 21, 80126, Naples, Italy

[†, ‡] Equally contributing authors

[*]Correspondence: andrea.bosso@unina.it (AB); Tel.: +39-081679129; Via Vicinale Cupa Cintia, 26, Department of Biology, University of Naples Federico II, 80126 Naples, Italy

A) **Nucleotide sequence of (P)FIBα- GVV27**
GATCCGGGAGTGGTCTGGGTTTCCTTTAGAGGGGCAGATTATTCCCTCAGGGCTGTTCGCATGAAAATTAGGCCCCTTGTGACCCAA
Amino acid sequence
DPGVVWVSFRGADYSLRAVRMKIRPLVTQ

B) **Nucleotide sequence of (P)FIBα-SFR22**
GATCCGTCCTTTAGAGGGGCAGATTATTCCCTCAGGGCTGTTCGCATGAAAATTAGGCCCCTTGTGACCCAA
Amino acid sequence
DPSFRGADYSLRAVRMKIRPLVTQ

C) **Nucleotide sequence of (P)FIBβ-GVV28**
GATCCGGGTGTGGTGTGGATGAATTGGAAAGGCAGCTGGTATAGCATGCGTAAAATGAGCATGAAAATTCGTCCGTTTTTTCCGCAGCA
G
Aminoacid sequence
DPGVVWMNWKGSWYSMRKMSMKIRPFFPQQ

D) **Nucleotide sequence of (P)FIBβ-NWK23**
GATCCGAATTGGAAAGGCAGCTGGTATAGCATGCGTAAAATGAGCATGAAAATTCGTCCGTTTTTTCCGCAGCAG
Aminoacid sequence
DPNWKGSWYSMRKMSMKIRPFFPQQ

E) **Nucleotide sequence of (P)FIBγ-GII30**
GATCCGGGTATTATTTGGGCGACCTGGAAAACCCGTTGGTATAGCATGAAAAAAACCACCATGAAAATTATTCCGTTTAACCGTCTGACC
ATTGGC
Aminoacid sequence
DPGIIWATWKTRWYSMKKTTMKIIPFNRLTIG

F) **Nucleotide sequence of (P)FIBγ-TWK25**
GATCCGACCTGGAAAACCCGTTGGTATAGCATGAAAAAAACCACCATGAAAATTATTCCGTTTAACCGTCTGACCATTGGC
Aminoacid sequence

DPTWKTRWYSMKKTTMKIIPFNRLTIG

G) **Nucleotide sequence of (C)FIBγ-TWK25**

GATTGCACCTGGAAAACCCGTTGGTATAGCATGAAAAAAACCACCATGAAAATTATTCCGTTTAACCGTCTGACCATTGGC

Aminoacid sequence

DCTWKTRWYSMKKTTMKIIPFNRLTIG

Figure S1. **Nucleotide and amino acid sequences of the recombinant peptides.** Blue: peptide; red: DP or DC cleavage sites.

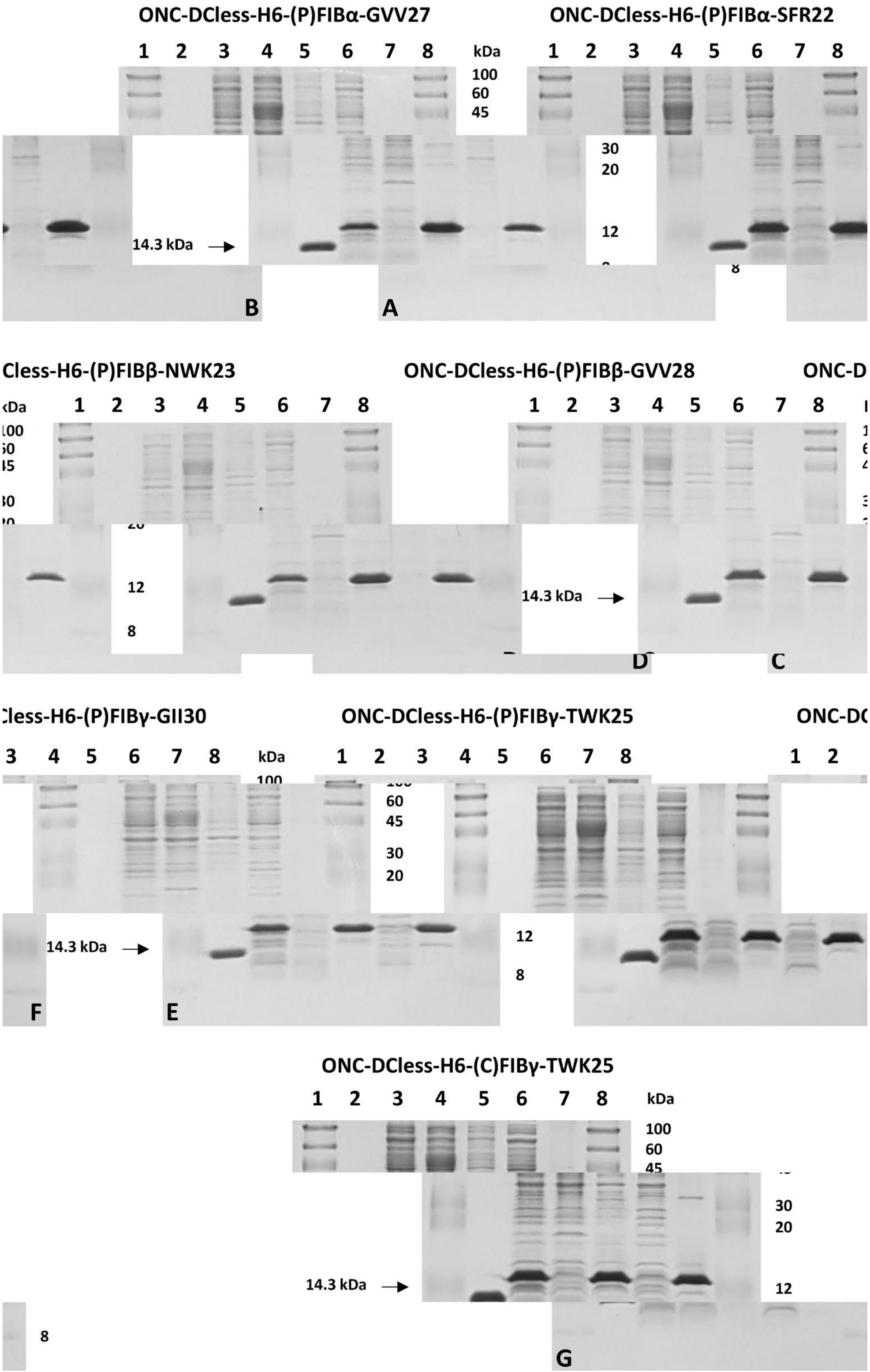

Figure S2. Expression and purification of the recombinant proteins. SDS-PAGE (15%) analysis of ONC-DCless-H6-(P)FIBα-GVV27 (A), ONC-DCless-H6-(P)FIBα-SFR22 (B), ONC-DCless-H6-(P)FIBβ-GVV28 (C), ONC-DCless-H6-(P)FIBβ-NWK23 (D), ONC-DCless-H6-(P)FIBγ-GII30 (E), ONC-DCless-H6-(P)FIBγ-TWK25 (F), ONC-DCless-H6-

(C)FIBγ-TWK25 (G). Lanes 1,8: molecular markers (8-12-20-30-45-60-100-220 kDa proteins); lane 2: *Gallus gallus* lysozyme (14.3 kDa); lanes 3,4: cellular lysates of induced and non-induced cultures, respectively; lanes 5,6: insoluble and soluble fractions after cell lysis, respectively; lane 7: uncleaved purified fusion proteins.

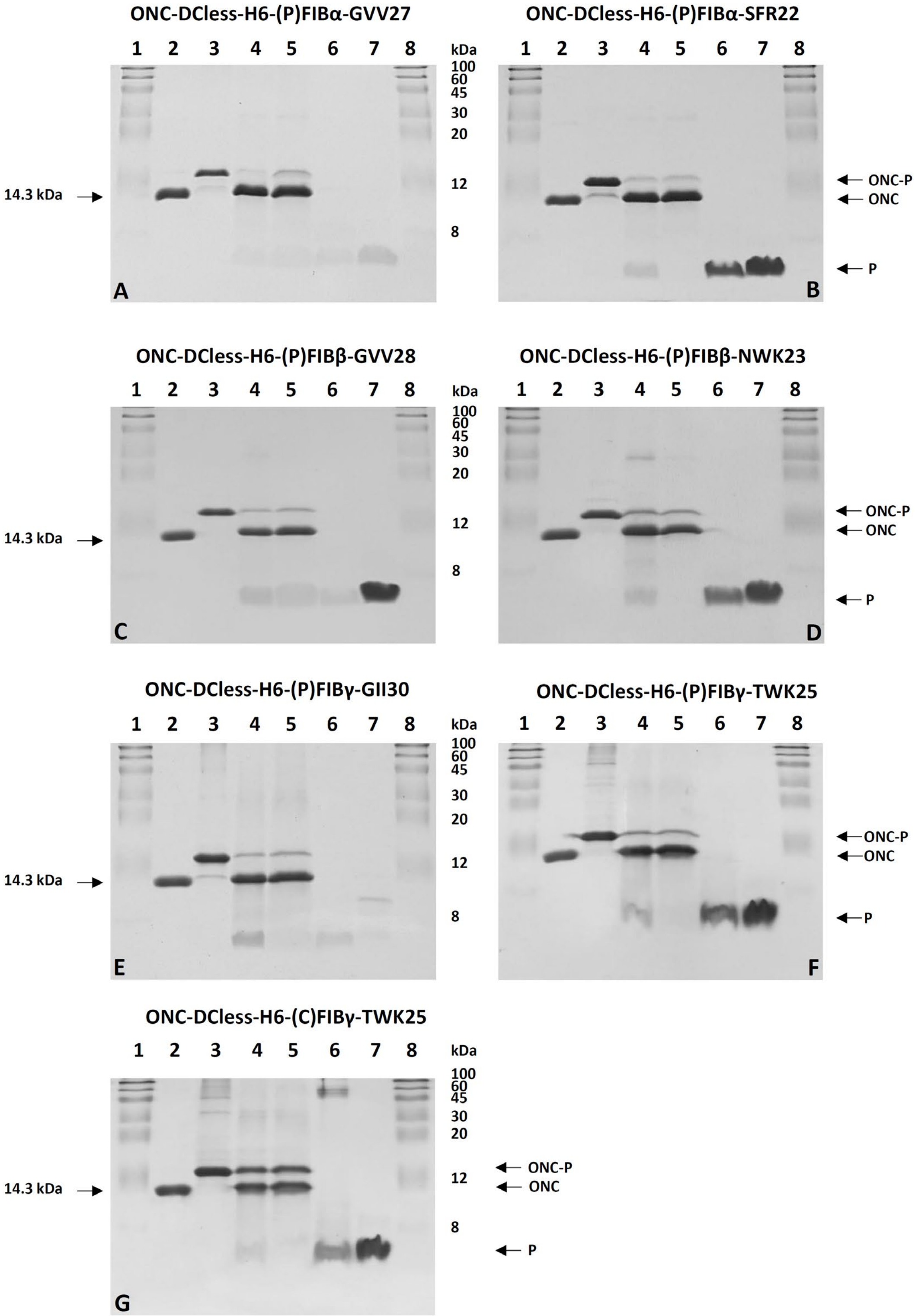

Figure S3. Hydrolysis and selective precipitation of carriers and fusion proteins. SDS-PAGE (20 %) analysis of chemical cleavage carried out at 60°C for 24 h at pH 2 in the presence of TCEP. (A) ONC-DCless-H6-(P)FIBα-GVV27, (B) ONC-DCless-H6-(P)FIBα-SFR22, (C) ONC-DCless-H6-(P)FIBβ-GVV28, (D) ONC-DCless-H6-(P)FIBβ-NWK23, (E) ONC-DCless-H6-(P)FIBγ-GII30, (F) ONC-DCless-H6-(P)FIBγ-TWK25, (G) ONC-DCless-H6-(C)FIBγ-TWK25.

Lanes 1,8: molecular markers (8-12-20-30-45-60-100-220 kDa proteins); lane 2: *Gallus gallus* lysozyme (14.3 kDa); lane 3: uncleaved purified fusion proteins; lane 4: fusion proteins after chemical cleavage; lanes 5,6: insoluble and soluble fractions after selective precipitation (pH 7.2 for 1 h at 25°C). Lane 7: RP-HPLC purified peptide. ONC-P: Onconase/Peptide fusion protein; ONC: Onconase carrier; P: peptide.

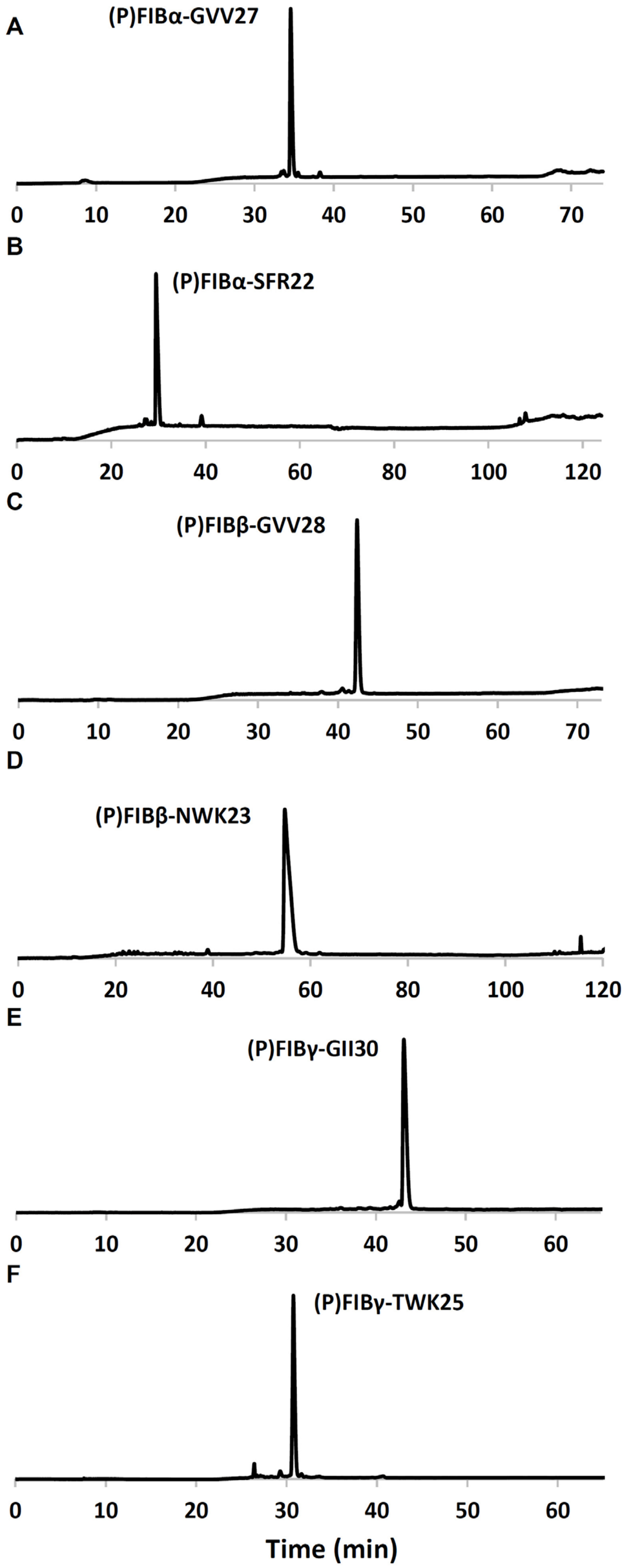

Figure S4. Peptide purity analyses. Peptides were analyzed by RP-HPLC carried out on Europa Protein 300 C18 column. Chromatograms were recorded at 280 nm. A) (P)FIBα-GVV27 (gradient 3); B) (P)FIBα-SFR22 (gradient 1); C)

(P)FIBβ-GVV28 (gradient 3); D) (P)FIBβ-NWK23 (gradient 1); E) (P)FIBγ-GII30 (gradient 3); F) (P)FIBγ-TWK25 (gradient 3).

Figure S5. Analysis of Raw 264.7 cell viability upon treatment with FIB-derived peptides and (P)GKY20 for (A) 6 and (B) 24 hours. Cell viability was determined by the MTT assay.

Figure S6. Analysis of the nitric oxide (NO) release by Raw 264.7 treated with 10 or 20 µM of each FIB-derived peptide or (P)GKY20 compared to LPS-administrated and untreated cells (CTRL).

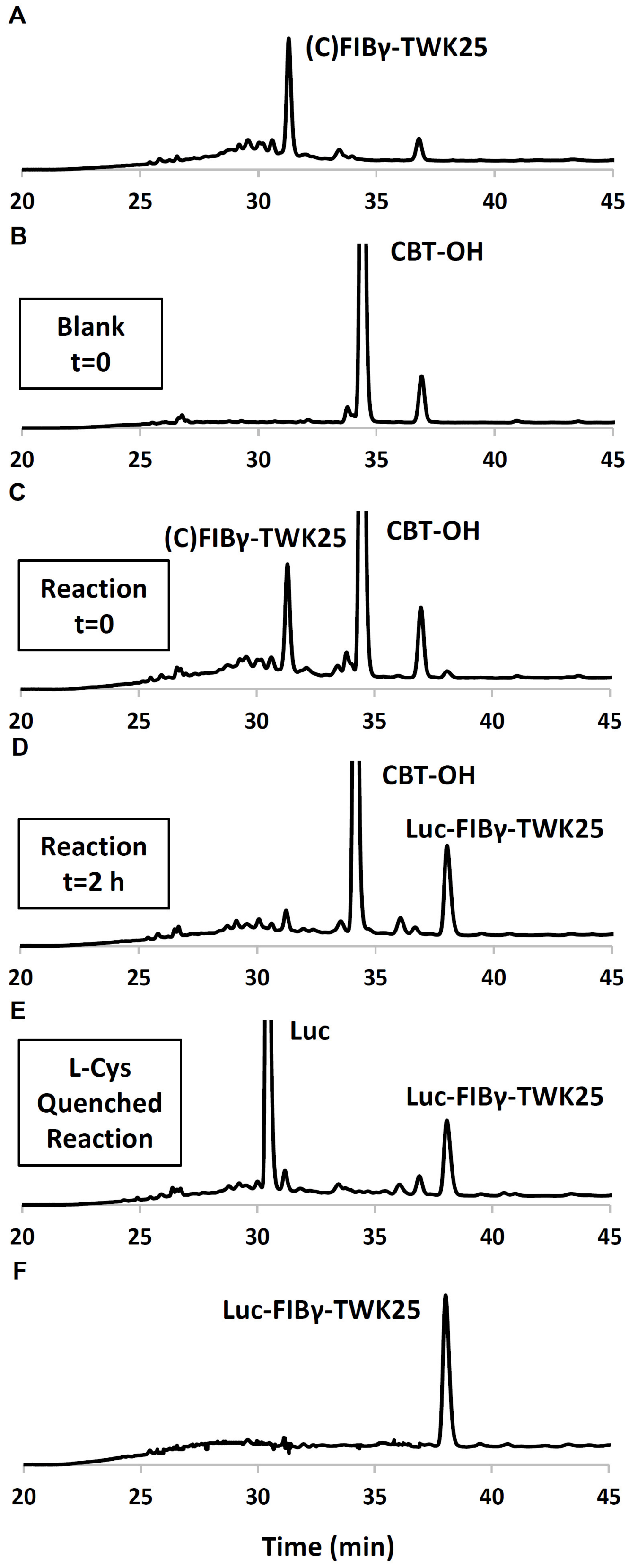

Figure S7. Preparation of Luc-FIBγ-TWK25. Condensation of (C)FIBγTWK25 with CBT-OH was monitored by RP-HPLC. Analyses were carried out on Europa Protein 300 C18 column by gradient 3. Chromatograms were recorded at 280 nm. A) (C)FIBγTWK25 peptide released by chemical hydrolysis of purified protein. B) Blank reaction carried out in absence of peptide (t=0). C) Condensation reaction at the beginning of reaction (t=0). D) Condensation reaction after 2 h

incubation (t=2 h). E) Condensation reaction after quenching with L-cysteine. F) Luc-FIBγ-TWK25 after RP-HPLC purification. Luc: luciferin.

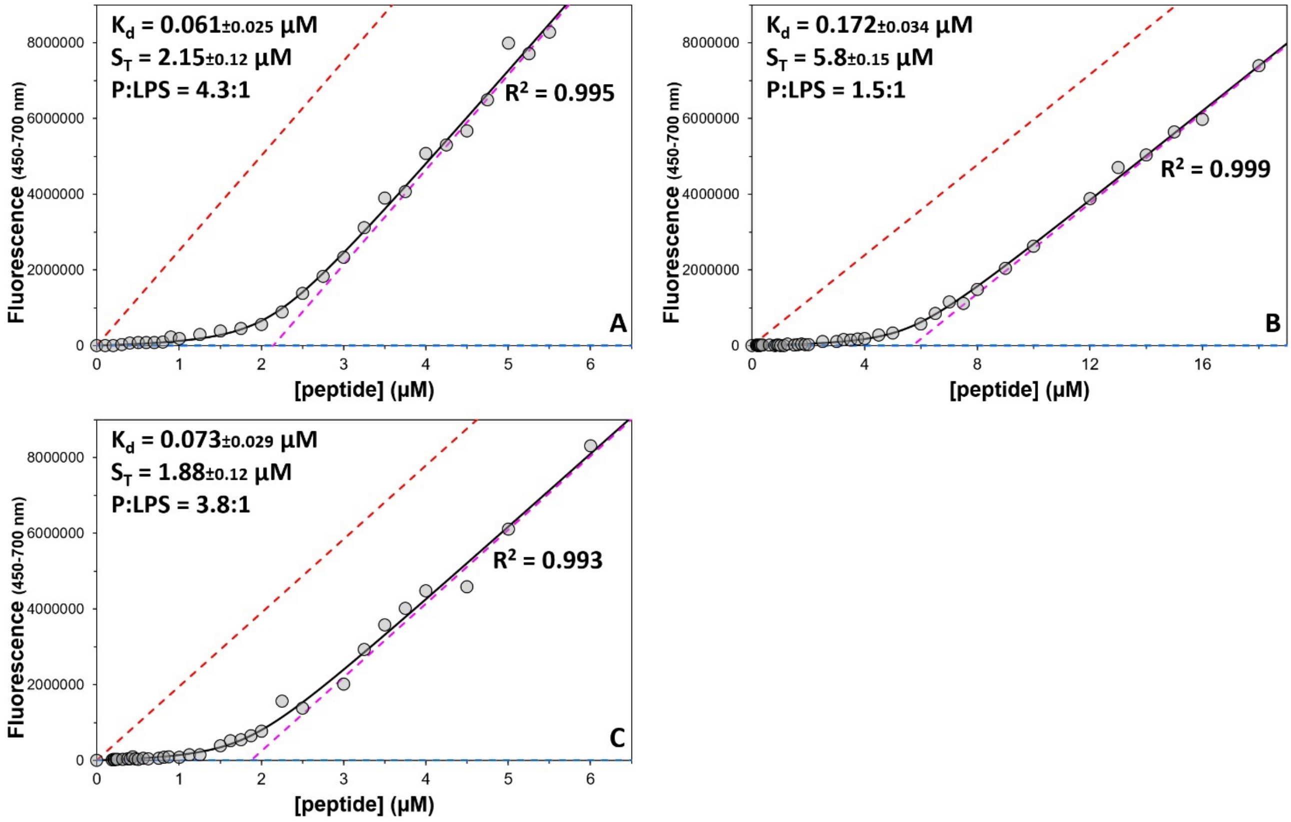

Figure S8. Determination of the K_d values and stoichiometry for the interaction of Luc-FIBγ-TWK25 with *E. coli* and *P. aeruginosa* LPS. (**A**) *E. coli* LPS (5 µg/mL). (**B**) *E. coli* LPS (40 µg/mL). (**C**) *P. aeruginosa* LPS (5 µg/mL). Black lines, K_d and stoichiometry (S_T) values were obtained by fitting fluorescence data as described in Methods section. The magenta dashed line is the asymptote of the black curve. Red and blue dashed lines are the expected fluorescence of the free and bound peptide, respectively. The ratio P:LPS was calculated from S_T, assuming that *E. coli* and *P. aeruginosa* LPS have an average molecular weight of 10 kDa.

Figure S9. Analysis of Vero cells viability upon treatment with FIB-derived peptides and (P)GKY20 for 24 hours. Cell viability was determined by the MTT assay.

Table S1. Extinction coefficients of recombinant proteins and peptides calculated by the ProtParam tool.

**Proteins**	**ε_{280} M^{-1} cm^{-1}**	**ε_{280} $^{(0.1\%)}$ mg/mL**
ONC-DCless-H6-(P)FIBα-GVV27	22,920	1.385
ONC-DCless-H6-(P)FIBα-SFR22	17,420	1.088
ONC-DCless-H6-(P)FIBβ-GVV28	33,920	2.001
ONC-DCless-H6-(P)FIBβ-NWK23	28,420	1.735
ONC-DCless-H6-(P)FIBγ-GII30	33,920	1.986
ONC-DCless-H6-(P)FIBγ-TWK25	28,420	1.718
ONC-DCless-H6-(C)FIBγ-TWK25	28,420	1.717
**Peptides**		
(P)FIBα-GVV27	6,990	2.182
(P)FIBα-SFR22	1,490	0.560
(P)FIBβ-GVV28	17,990	4.991
(P)FIBβ-NWK23	12,490	4.120
(P)FIBγ-GII30	17,990	4.811
(P)FIBγ-TWK25	12,490	3.904
(C)FIBγ-TWK25	12,490	3.897
(P)GKY20	8,480	3.250

Table S2. RP-HPLC gradients.

**Peptide**	**Gradient number[1]**		
	**Cleavage analysis**	**Purification**	**Purity analysis**
(P)FIBα-GVV27	1	1	3
(P)FIBα-SFR22	2	2	1
(P)FIBβ-GVV28	3	4	3
(P)FIBβ-NWK23	1	1	1
(P)FIBγ-GII30	3	4	3
(P)FIBγ-TWK25	1	1	1
(C)FIBγ-TWK25	1		
Luc-FIBγ-TWK25	-	1	3

[1]The solvents were 0.05% trifluoracetic acid (TFA) in water (solvent A) and 0.05% TFA in acetonitrile (solvent B).

Elution profiles were recorded at 280 nm wavelength at a flow rate of 2 ml/min by linear gradients as follows:

Gradient 1: from 5% to 25% solvent B in 10 min, from 25% to 29% solvent B in 60 min, isocratic elution at 29% solvent B for 20 min, from 29% to 60% solvent B in 20 min, from 60% to 100% solvent B in 5 min, isocratic elution at 100% B for 10 min.

Gradient 2: from 5% to 20% solvent B in 10 min, from 20% to 25% solvent B in 60 min, isocratic elution at 25% solvent B for 20 min, from 25% to 60% solvent B in 20 min, from 60% to 100% solvent B in 5 min, isocratic elution at 100% solvent B for 10 min.

Gradient 3: isocratic elution at 5% solvent B for 10 min, from 5% to 30% solvent B in 5 min, from 30% to 38% solvent B in 40 min, from 38% to 100% solvent B in 10 min, isocratic elution at 100% solvent B for 10 min.
Gradient 4: from 5% to 27% solvent B in 10 min, from 27% to 31% solvent B in 60 min, isocratic elution at 31% solvent B for 60 min, from 31% to 100% solvent B in 20 min, isocratic elution at 100% solvent B for 20 min.
